# Antimicrobial Polymers at the Membrane Interface: Impact of Macromolecular Architecture

**DOI:** 10.1002/smll.202406534

**Published:** 2024-12-30

**Authors:** Alain M. Bapolisi, Anne‐Catherine Lehnen, Rainhard Machatschek, Gaetano Mangiapia, Eric Mark, Jean‐Francois Moulin, Petra Wendler, Stephen C. L. Hall, Matthias Hartlieb

**Affiliations:** ^1^ Institute of Chemistry University of Potsdam Karl‐Liebknecht‐Straße 24‐25 14476 Potsdam Germany; ^2^ Fraunhofer Institute for Applied Polymer Research (IAP) Geiselbergstraße 69 14476 Potsdam Germany; ^3^ Institute of Active Polymers Helmholtz‐Zentrum Hereon Kantstraße 55 14513 Teltow Germany; ^4^ German Engineering Materials Science Centre (GEMS) am Heinz Maier‐Leibnitz Zentrum (MLZ) Helmholtz‐Zentrum Hereon Lichtenbergstr. 1 85748 Garching bei München Germany; ^5^ Institute of Biochemistry and Biology Department of Biochemistry University of Potsdam Karl‐Liebknecht Strasse 24‐25 14476 Potsdam Germany; ^6^ ISIS Neutron and Muon Source Rutherford Appleton Laboratory Didcot OX11 0QX UK

**Keywords:** antimicrobial polymers, bottlebrush copolymers, Langmuir monolayer, neutron reflectivity, polymer–membrane interactions

## Abstract

Antimicrobial resistance (AMR) is a major cause of death worldwide. This urges the search for alternatives to antibiotics, and antimicrobial polymers hold promise due to their reduced susceptibility to AMR. The topology of such macromolecules has a strong impact on their activity, with bottlebrush architectures outperforming their linear counterparts significantly. Consequently, understanding the specific behavior of macromolecules featuring a confined conformation of linear subunits is pertinent. This study focusses on revealing fundamental differences between architectures regarding properties as well as interaction with biological membranes. Various analytical techniques (using membrane mimics and spectroscopic methods) are used to generate insights revealing the following trends: A) The reduction of degrees of freedom in bottle brushes reduces their tendencies for self‐assembly and undesired protein interaction. B) When compared to linear polymers, bottlebrushes attach to membranes faster and more efficiently as well as in a unimolecular fashion. Their multivalent presentation of linear subunits also leads to aggregation between liposomes, which is not induced by linear polymers. C) Neutron reflectometry measurements show an increased tendency of bottle brushes to insert into the hydrophobic tails of phospholipid monolayers. The knowledge about these features will fuel the future development of even more efficient antimicrobial polymers.

## Graphical abstract

Important differences in the solution behavior and membrane interaction of cationic linear and bottle brush copolymers are elucidated, with these properties linked to their antimicrobial behavior. Different types of model membranes and their interaction with polymers are investigated using various analytical techniques including neutron reflectivity. Culminated data highlight the importance of polymer architecture and the advantages of bottle brush polymer.

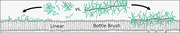

## 1 Introduction

The rapid and alarming emergence of antimicrobial resistance (AMR) as one of the leading causes of death worldwide is pushing researchers to find new antimicrobials with potent activity, lower toxicity and most importantly, mechanisms of action that are less susceptible to AMR development.^[^ ^1^, ^2^ ^]^ Antimicrobial polymers (APs) are one of the most promising materials in that regard. Similar to the antimicrobial peptides that they mimic, the amphiphilic balance between cationic and hydrophobic units is believed to be the most important feature regulating membrane activity. By electrostatic interactions, cationic groups can bind to the negatively charged bacterial membrane while hydrophobic units can insert into the membrane resulting in membrane permeabilization and cell death.^[^ ^3^, ^4^, ^5^, ^6^, ^7^ ^]^

In addition to amphiphilicity, properties of APs such as molecular weight,^[^ ^8^ ^]^ charge density,^[^ ^9^ ^]^ spatial arrangement,^[^ ^10^, ^11^ ^]^ and stereochemistry of subunits,^[^ ^12^ ^]^ as well as overall polymer architecture^[^ ^13^, ^14^, ^15^, ^16^ ^]^ were proven to influence cytotoxicity and selective action toward bacterial membranes. State of the art polymerization techniques permit the synthesis of various APs of different chemical compositions and topologies;^[^ ^17^, ^18^ ^]^ ranging from linear statistical/random or block polymers^[^ ^19^, ^20^, ^21^, ^22^ ^]^ to more complex architectures such as star‐shaped,^[^ ^13^, ^15^ ^]^ hyperbranched,^[^ ^13^, ^14^ ^]^ or bottlebrush copolymers.^[^ ^23^, ^24^ ^]^

However, thorough investigations of APs mechanisms of action are still required, as those can be very specific depending not only on the chemical properties and topology of the polymers but also on the particularities of the targeted microorganisms’ membrane. The complex structural organization and composition of bacterial cell envelopes containing native phospholipids, lipopolysaccharides, peptidoglycans, lipoteichoic acids, and proteins varies between species,^[^ ^25^ ^]^ which hinders the development of a more generalized understanding of the modes of action of APs at the membrane interface. Here, artificial membrane models such as large and giant unilamellar vesicles or supported bi‐ and mono‐layers imitating bacterial membranes’ composition, can be used as simplistic and controllable models to study such interactions on the molecular level.^[^ ^26^, ^27^, ^28^, ^29^, ^30^, ^31^, ^32^ ^]^

**Scheme 1:**
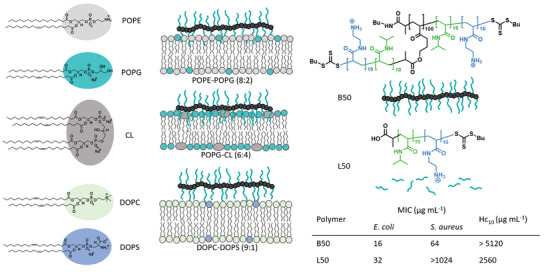
Phospholipids structures and schematic representation of interaction of bottlebrush APs with different membrane models composed of POPE‐POPG (8:2), POPG‐CL (6:4) and, DOPC‐DOPS (9:1) mimicking inner membrane of *E. coli*, *S. aureus*, and red blood cell respectively; Structures and schematic representation of APs with improved aspect ratio and side chain grafting density as well as bioactivity data.

Correlating the physico‐chemical properties of polymers to their antibacterial activity by means of membrane models or artificial cells imitating the lipid composition of bacterial membranes has proven to be instrumental in understanding the polymer–membrane interface.^[^ ^33^, ^34^ ^]^ We previously reported on efforts to probe the impact of polymer architecture on antimicrobial activity by combining analytical techniques with membrane models to understand the differences between linear and bottlebrush APs made of statistically distributed cationic amino ethyl acrylamide (AEAm) and hydrophobic *N*‐isopropyl acrylamide (NiPAm) units. The study showed that the advantageous physico‐chemical properties of these bottlebrush polymers, in particular their multivalence and their more compact conformation, were determinant factors supporting their improved antimicrobial activity compared to linear counterparts.^[^ ^35^ ^]^ However, the grafting‐through approach used to synthesize these bottlebrush APs via ring‐opening metathesis polymerization (ROMP) of norbornene end groups of linear side chains^[^ ^23^ ^]^ did not enable straightforward control over side chain length or density of bottlebrushes. To further develop this type of APs, we envisaged improving their confinement by independently varying grafting density of side chains and the backbone length. This was recently achieved by a grafting‐from approach with two subsequent reversible addition‐fragmentation chain transfer (RAFT) polymerizations resulting in bottlebrush APs with higher aspect ratio (**Scheme** **1**).^[^ ^36^ ^]^ The first RAFT polymerization was used to produce the backbone (DP = 100) while the second polymerization allowed grafting of short side chains (graft DP = 20) made of NiPAm and AEAm copolymers with variable cationic density. As expected, the new bottlebrush copolymers series generally outperformed their linear analogues in antimicrobial activity. Remarkably, the bottlebrush APs with 50% cationic monomer (B50) proved to be the best performer and more selective toward the tested bacteria strains (*Escherichia coli, Pseudomonas aeruginosa*, and Methicillin‐resistant *Staphylococcus aureus* (MRSA)) over red blood cells when compared to their linear counterpart (L50 which also possesses a 50% cationic monomer content). Surprisingly, L50 and B50 had comparable minimum inhibition concentration (MIC) against *E. coli* (with MIC of 32 and 16 µg mL^−1^, respectively) but the linear L50 showed no inhibition of MRSA strains at tested concentrations (MIC > 1024 µg mL^− 1^) whereas B50 still proved to be very active (MIC = 64 µg mL^−1^) against the gram positive strain.^[^ ^36^ ^]^ This was particularly surprising as it was previously reported that gram positive strains discriminate against larger polymers, which was attributed to an entrapment in the thick outer peptidoglycan layer via entanglements.^[^ ^37^ ^]^ Apparently, the compact nature of bottlebrushes^[^ ^38^ ^]^ prevents entanglement in the outer cell wall and enables efficient membrane activity.

To determine whether there are also other factors at play, we investigate the impact of polymer architecture of APs on membrane interaction. Various techniques were used to gain an in depth understanding of the physico‐chemical behavior of APs in aqueous media and at the membrane interface. To this end, antimicrobial bottlebrush copolymers with high aspect ratio and their linear analogues were studied regarding their interaction with different membrane models.

## 2 Results and Discussion

### 2.1 Physico‐Chemical Characterization

**Figure 1:**
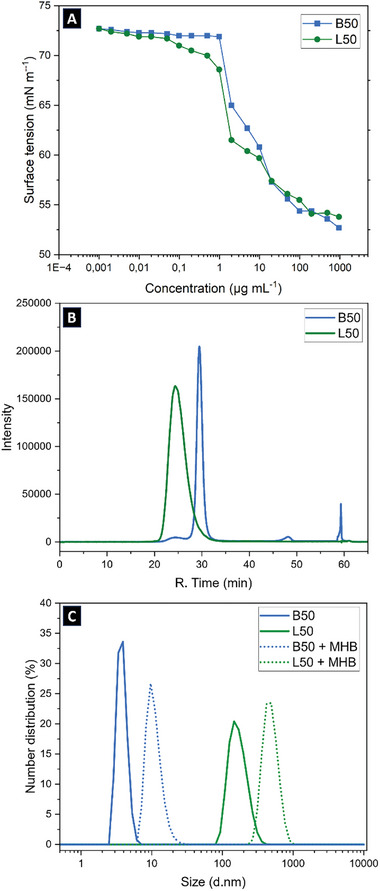
A) Comparative aqueous behavior of linear (L50 in green) and bottlebrush (B50 in blue) APs by tensiometric determination of surface activity of polymers. B) HPLC spectra measured with gradient elution from water to acetonitrile on a C18 column. C) Hydrodynamic size distribution of polymers (solid lines) and formation of polymer‐protein complex (dot lines) with Müller Hinton Broth (MHB) by dynamic light scattering.

A key requisite for antimicrobial activity is the ability to adsorb, and interact with interfaces in aqueous environments, which is benchmarked by the amphiphilicity of the antimicrobial agent. In a first step, the interfacial activity of B50 and L50 was determined by measuring their surface tension at the air–liquid interface upon increasing polymer concentrations (from 0 to 1000 µg mL^−1^) in PBS at simulated extracellular condition (pH 7). As depicted in **Figure** **1**A, the two polymers imparted similar changes in surface tension as a function of concentration. At low polymer concentrations, there is no effect on the surface tension of water (72 mN m^−1^). Upon increasing the polymer concentration, the interface gets crowded leading to a drastic change in surface tension. When the interface is saturated with the polymer, additional molecules do not lead to a further increase in polymer surface concentration and a plateau can be observed around 54 mN m^−1^. Overall, despite their different topologies, L50 and B50 are equally surface active. We infer that the interfacial activity is mainly determined by the amphiphilicity of the polymers, which presumably is defined by their (equal) composition. The similar charge density of L50 and B50 was further confirmed (at concentrations of 1 mg mL^−1^) with similar zeta potential values of (54 ± 6) and (53 ± 9) mV for B50 and L50, respectively.

The degree of protonation of membrane active antimicrobial polymers can influence their biological activity as their cationic charge is important for electrostatic binding with negatively charged bacteria membranes. Both investigated APs have pK_a_ values > 7.4 and would remain protonated at physiological pH and consequently be membrane active. However, L50 has a somewhat higher pK_a_ value of 8.8 compared to B50 with pK_a_ = 7.9 (Figure S1, Supporting Information), meaning that L50 is only deprotonated in more basic conditions. The pK_a_ is apparently diminished for B50 due to compact conformation of bottlebrush structures resulting in increased density of amino/ammonium groups and eventually electrostatic repulsion of protonated functionalities.^[^ ^34^, ^39^ ^]^ This is consistent with our previously reported data on copolymers having the same monomer composition (e.g., NiPAAm and AEAm) for which the pK_a_ of the linear form (pK_a_ = 8.0) was found to be higher than the pK_a_ of the bottlebrush form (pK_a_ = 7.5).^[^ ^35^ ^]^ While the zeta potential suggests little difference in the degree of protonation, the difference in pK_a_ could become more important when the pH value deviates from ideal physiological conditions. In fact, among the environmental variables that control the metabolism of bacteria, pH is an important factor particularly regarding their growth and survival. While common bacteria strains are neutrophiles, i.e., optimally grow and survive at neutral microenvironment (5 < pH < 9), a few bacteria are also known to be acidophiles (pH < 5) or some even alkaliphiles (pH > 9).^[^ ^40^ ^]^ Hence, depending on the bacterial strains, APs may be deprotonated and consequently become less active when the bacterial microenvironment gets more alkaline.

Despite the similar surface activity and cation content, the two polymers still showed different selectivity and cytotoxicity profiles.^[^ ^36^ ^]^ Hydrophobicity is known as one of the main factors influencing the selectivity of APs for bacteria over mammalian cells.^[^ ^41^, ^42^ ^]^ Reverse phase high performance liquid chromatography (RP‐HPLC) can be used to discriminate polymers of different composition or architecture by comparing elution times which can be correlated to hydrophobicity.^[^ ^43^, ^44^ ^]^

We performed RP‐HPLC measurements on our two polymers in aqueous solution (5 mg mL^−1^) with gradient elution from water to acetonitrile solvents system on a C18 column, i.e., a shorter retention time would correspond to a less hydrophobic substance. Results indicate that B50 was more hydrophobic compared to the linear L50 (Figure 1B). The apparent higher hydrophobicity of bottlebrush APs could also explain their hemolytic behavior and cytotoxicity to HEK cells previously observed in bioassays compared to their linear counterparts.^[^ ^36^ ^]^ This difference in hydrophobicity could be attributed to the densely packed architecture. As previously demonstrated for bottlebrush copolymers with similar monomer composition in their side chains,^[^ ^35^ ^]^ B50 is expected to have more intramolecular hydrogen bonding. In consequence, the molecules are less solvated and stay rigid with no solvent shielding effect while having hydrophobic and cationic units equally exposed. In contrast, linear polymers can bury their hydrophobic units within the coil or within supramolecular assemblies to optimize their energy of solvation in aqueous environments and thereby appear more hydrophilic.

**Scheme 2:**
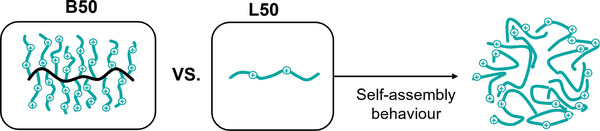
Illustration of self‐ assembly behavior of linear copolymers tending to shield and hide their hydrophobic units in aqueous medium resulting in decreased hydrophobicity and increased hydrodynamic size due to cationic group exposition at the shell.

Dynamic light scattering (DLS) measurements further supported this shielding of hydrophobicity by linear APs. As shown in Figure 1C, L50 appears to be forming structures of considerable hydrodynamic radii that could be related to self‐assembly in water (milliQ) as well as in PBS (Figure S8, Supporting Information) whilst B50 showed a small hydrodynamic radius of narrow distribution, likely corresponding to unimolecular species. This confirms the formation of supramolecular structures for L50 while B50 molecules remain isolated (**Scheme** **2**). It should be noted that while relatively high concentrations of 1 mg mL^−1^ were used to enable DLS measurements with good signal to noise ratio, changes in interfacial tension at much lower concentrations suggest that such a behavior could also be expected at lower concentrations that are relevant in a biological context.

The considerable hydrodynamic size of L50 could also explain the generally higher MIC values previously observed against tested bacteria (*E. coli, P. aeruginosa*, MRSA).^[^ ^36^ ^]^ In addition to the formation of supramolecular complexes, linear APs are also more likely to form protein‐polymer complexes (PPC) in Müller Hinton Broth (MHB) medium both of which reduces the effective AP concentration in the medium.^[^ ^45^, ^46^ ^]^ To confirm this, we dispersed the polymers in MHB and used DLS to probe the change in hydrodynamic size. The already self‐assembled L50 in water showed a considerable increase in hydrodynamic radius in MHB medium (Figure 1C), suggesting a stronger interaction with proteins compared to B50.

### 2.2 Interaction with Membrane Models

We recently investigated the polymer–membrane interaction using artificial membrane models mimicking membranes of bacteria or mammalian cells.^[^ ^35^, ^36^, ^47^ ^]^ A phospholipid mixture made of 80% (weight ratio) POPE (2‐Oleoyl‐1‐palmitoylsn‐glycero‐3‐phosphoethanolamine) and 20% POPG (2‐Oleoyl‐1‐palmitoyl‐sn‐glycero‐3‐phospho‐rac‐(1‐glycerol) sodium salt) was used to imitate *E. coli* membranes,^[^ ^22^, ^37^ ^]^ 60% POPG and 40% CL (18:1 Cardiolipin) for *S. aureus* membranes,^[^ ^47^, ^48^, ^49^ ^]^ and 90% DOPC (1,2‐dioleoyl‐sn‐glycero‐3‐phosphocholine) and 10% DOPS (1,2‐dioleoyl‐sn‐glycero‐3‐phospho‐L‐serine (sodium salt)) to reflect RBCs (red blood cells) membranes (Scheme 1).^[^ ^12^ ^]^ The reported dye leakage experiments using these membrane models were consistent with the bioassays results and further supported the higher selectivity of B50 to *S. aureus*.^[^ ^36^ ^]^ These tests hinted that the observed selectivity is a direct result of the molecular membrane composition and the interaction profile of the different polymers with the interface.

**Figure 2:**
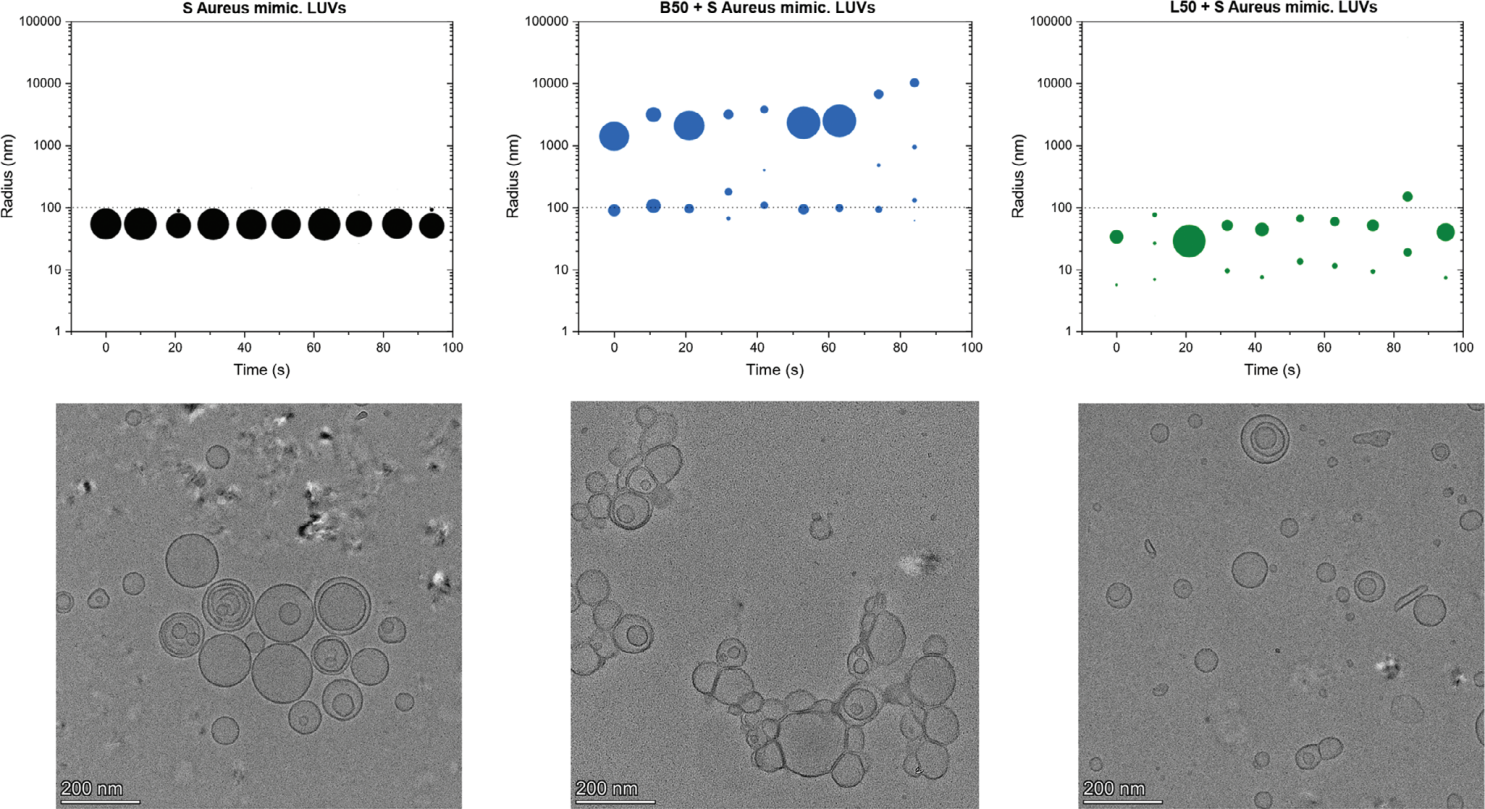
DLS size distribution (top) and respective Cryo‐EM images (bottom) of *S. aureus* mimicking large unilamellar vesicles (0.5mg mL^−1^) alone (on the left) and incubated with APs (512 µg mL^−1^) of bottlebrush topology (B50 in the middle) resulting in more aggregation of vesicles, and linear copolymers (L50 on the right) with less aggregation observed. Radius values for DLS represent the hydrodynamic mean size distribution by intensity of the LUVs and the circles represent the normalized amplitudes of respective distributions recorded at each measurement as a function of time (number of measurements = 10).

**Figure 3:**
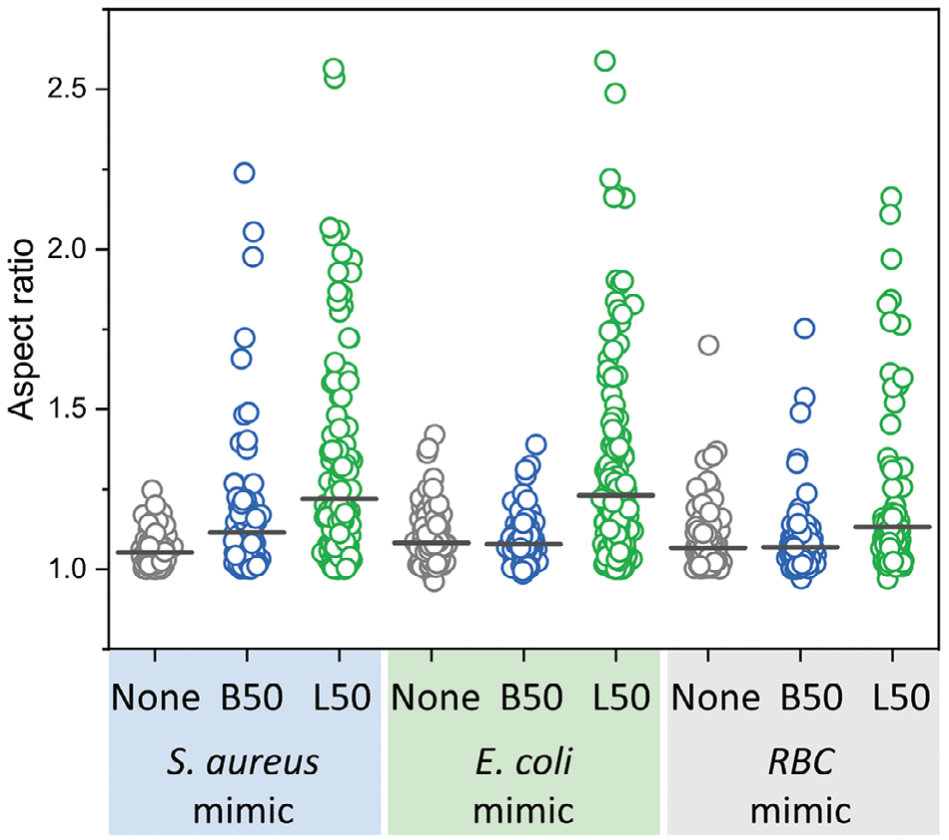
Aspect ratio distribution derived from cryo‐EM images of Large Unilamellar vesicles (LUVs) with lipid composition mimicking *S. aureus* (SA_LUVs), *E. coli* (EC_LUVs), and red blood cells (RBC_LUVs). LUVs alone (as control in grey) are compared to LUVs incubated with either linear APs (L50 in green) or bottlebrush APs (B50 in blue). The aspect ratio was obtained by dividing the measured length to width of isolated LUVs (at least 150 vesicles for each series) measured using Fiji software. A perfect circular vesicle will have an aspect ratio of 1. Horizontal lines (black) in each series represent mean values of the aspect ratio distribution.

These interactions at the polymer–membrane interface are studied here using the same membrane mimicking vesicles in greater detail by extended analytical techniques. Large unilamellar vesicles (LUVs) were incubated with polymers and analyzed with Xtral DLS and cryogenic electron microscopy (cryo‐EM). Surprisingly, B50 induced aggregation of vesicles of all membranes’ models tested (e.g., LUVs mimicking *S. aureus, E. coli*, and RBC) whereas for linear L50 a decrease in size of the vesicles was observed by DLS (**Figures** **2** and S2, Supporting Information). The pronounced aggregation tendency induced by B50 compared to L50 on *S. aureus* mimicking LUVs was confirmed by cryo‐EM images where huge aggregates of linked vesicles could be observed for B50 (Figures 2 and S6, Supporting Information). However, aggregation differences could not be evidenced by cryo‐EM images on LUVs mimicking *E. coli* and RBC (Figure S6, Supporting Information). Besides the aggregation behavior, changes in the shape of vesicles induced by both polymers were observed on isolated vesicles (excluding aggregation regions) from cryo‐EM images. In comparison to B50, the L50 polymer induced a remarkable change in aspect ratio of LUVs on all the three membrane models (**Figure** **3**). This indicates that short linear statistical co‐polymers (with degree of polymerization (DP) of 20), already proven to be forming micelles‐like assemblies in aqueous media, are acting more like classical surfactant micelles and could interact with vesicles by disrupting the phospholipid bilayer and inducing subsequent leakage of vesicles resulting in smaller sizes and deformed shapes (aspect ratio) of LUVs.

It is worth mentioning that the previously investigated bottlebrush APs with longer side chains and shorter backbone did not induce aggregation, but rather change in aspect ratio of LUVs.^[^ ^35^ ^]^ This apparent difference in behavior of the two bottlebrush APs can be explained by their different topologies which was recently proven to be determinant in their interaction mechanism with bacteria.^[^ ^50^ ^]^ For densely packed bottlebrush configuration with a higher aspect ratio, like in the present system (backbone DP of 100 and side chain DP of 20), the mobility of side chains is systematically reduced. The stiffness and multivalence are therefore increased and could then promote linkage of neighboring vesicles through electrostatic interaction between the negatively charged vesicles and the rod‐like positively charged APs. While for bottlebrushes with short backbone and longer side chains (previously explored),^[^ ^35^ ^]^ a higher chain mobility is expected and the potential of unimolecular micellization is promoted, and such intramolecular rearrangement at the interface is less inclined to aggregation of vesicles.

**Figure 4:**
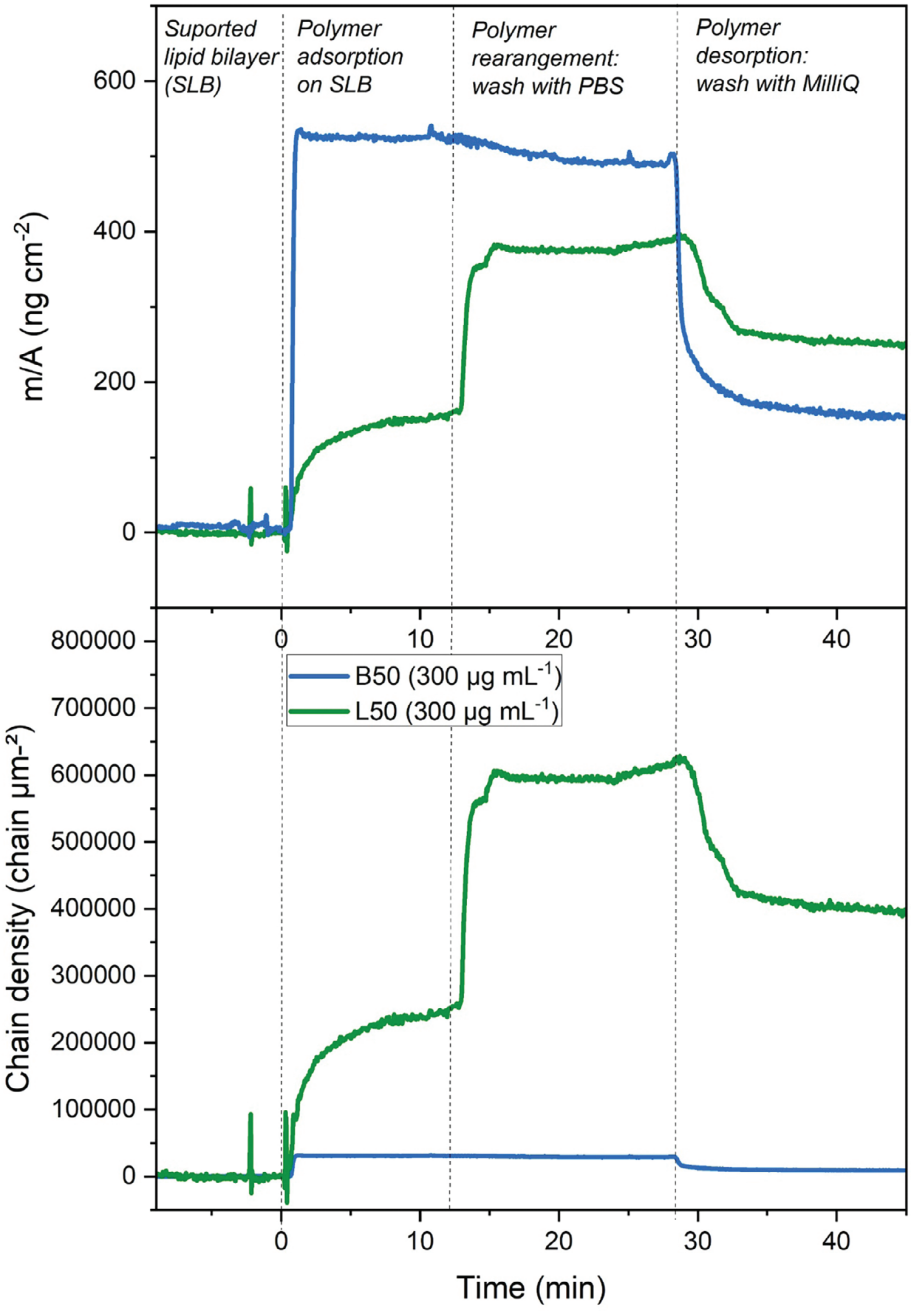
Mass per area (m/a) adsorption (top) and chain density (bottom) differences induced by linear L50 (in green) and bottlebrush B50 (in blue) on an initially formed supported lipid bilayer (SLB) composed of DOPC‐DOPS (9:1) lipids. The time of injection of polymer is considered as initial time (time = 0 min) and is used to normalize data fitted in Sauerbrey's equation (for details see experimental parts and Figure S5, Supporting Information). To obtain the chain density, molecular weights from size exclusion chromatography of L50 (Mn = 3800 g mol^−1^) and B50 (Mn = 102500 g mol^−1^) were considered.^[^ ^36^ ^]^

QCM‐D measurements on supported lipid bilayers (SLB), comprised of a DOPC‐DOPS (9:1 weight ratio) lipid system mimicking RBC allowed to study the binding kinetics of the two polymers, as depicted in **Figures** **4** and S5 (Supporting Information). As expected, both polymers adsorbed very fast on the SLB and the mass of polymers adsorbed per surface of SLB was higher for bottlebrush APs (B50) compared to the linear (L50). More molecules of L50 were adsorbed in comparison to bottlebrush considering their respective molecular weights. This is consistent with our previously reported investigations which demonstrated that the multivalence and compact conformation of APs in bottlebrush architecture and high molecular weight were resulting in higher mass adsorption as well as lower molecular chain density.^[^ ^35^ ^]^ However, after polymer adsorption, the PBS rinse surprisingly exposed the considerable structural rearrangement of L50 (further mass/chains adsorbed) which is not the case for B50. This could be explained by the assembly behavior of L50 molecules which are first binding to the SLB in a soft micellar‐like manner and then the rinsing with PBS induces a dispersion effect that disassembles the chains. The resulting individual linear chains subsequently rearrange to the interface thanks to their conformation flexibility leading to a denser polymer layer with considerable decrease in frequency (Δ*f*) and consequently an increase in mass adsorption. An additional rinse with MilliQ water induced the desorption of polymer which seems to be more sudden for bottlebrush considering their reduced chain mobility while linear chains are gradually desorbed.

**Figure 5:**
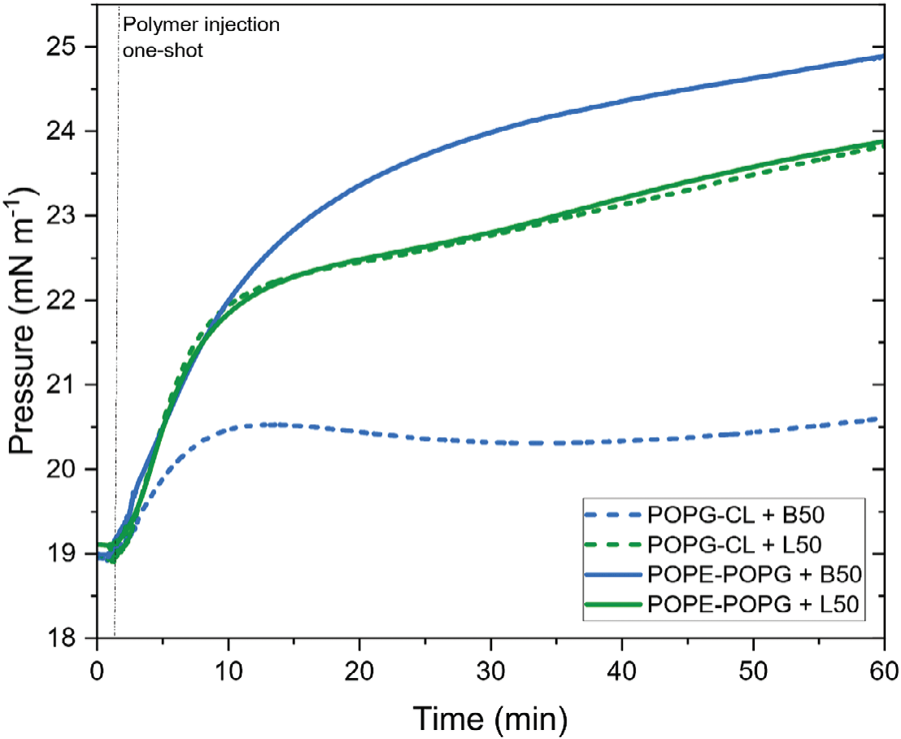
Surface pressure changes over time induced by single injections of polymers B50 (in blue) and L50 (in green), with final polymer concentration of 0.4 µg mL^−1^, under Langmuir monolayers earlier stabilized at 19 mN m^−1^ made of POPG‐CL (6:4 mass ratio) (small dash lines) and POPE‐POPG (8:2 mass ratio) (plain lines).

To further investigate the correlation between binding behavior and polymer architecture, Langmuir monolayers with lipid compositions mimicking *E. coli* (POPE‐POPG, 8:2 weight ratio) and *S. aureus* (POPG‐CL, 6:4 weight ratio) were used for interaction studies at a surface pressure of ≈19 mN m^−1^ which was the highest surface pressure where stable monolayers could be prepared. The lipid composition mimicking red blood cell (DOPC‐DOPS (9:1 weight ratio)) did not permit formation of the prerequisite stable monolayer and was therefore not considered for Langmuir assays. Injecting polymer solutions (40 µg mL^−1^) underneath the stabilized monolayers induced an increase in the surface pressure as depicted in **Figure** **5**. L50 showed very comparable adsorption kinetics and increase in surface pressure (∆π = 4.8 mN m^−1^ after 1 h) with both POPE‐POPG and POPG‐CL monolayers. This suggests that the linear polymers interact similarly with both monolayers, which is due to their conformational flexibility.

In contrast, there were marked differences in the adsorption of B50 to the two different lipid mixtures. In case of the POPE‐POPG monolayer, the pressure increased considerably (∆π = 5.8 mm m^−1^) whereas for the POPG‐CL monolayer, the increase in surface pressure was much lower (∆*π* = 1.6 mN m^−1^). The observation from single injections were consistent with the curves obtained upon stepwise increase of the polymer concentration underneath the monolayers (Figure S3, Supporting Information).

While these differences cannot be translated directly into MIC results, it is noteworthy that the adsorption behavior of the bottlebrush APs depends on the monolayer composition. This suggests a more specific interaction mechanism when compared to linear polymers and a potential pathway to enhanced selectivity for APs.

The Langmuir monolayers alone and with the adsorbed polymers after the experiments shown in Figure 5 were transferred onto silicon wafers using the Langmuir–Schäfer method. This transfer exposes the water‐side of the layers where the headgroups of the lipids and most likely also the adsorbed polymers are located.

**Figure 6:**
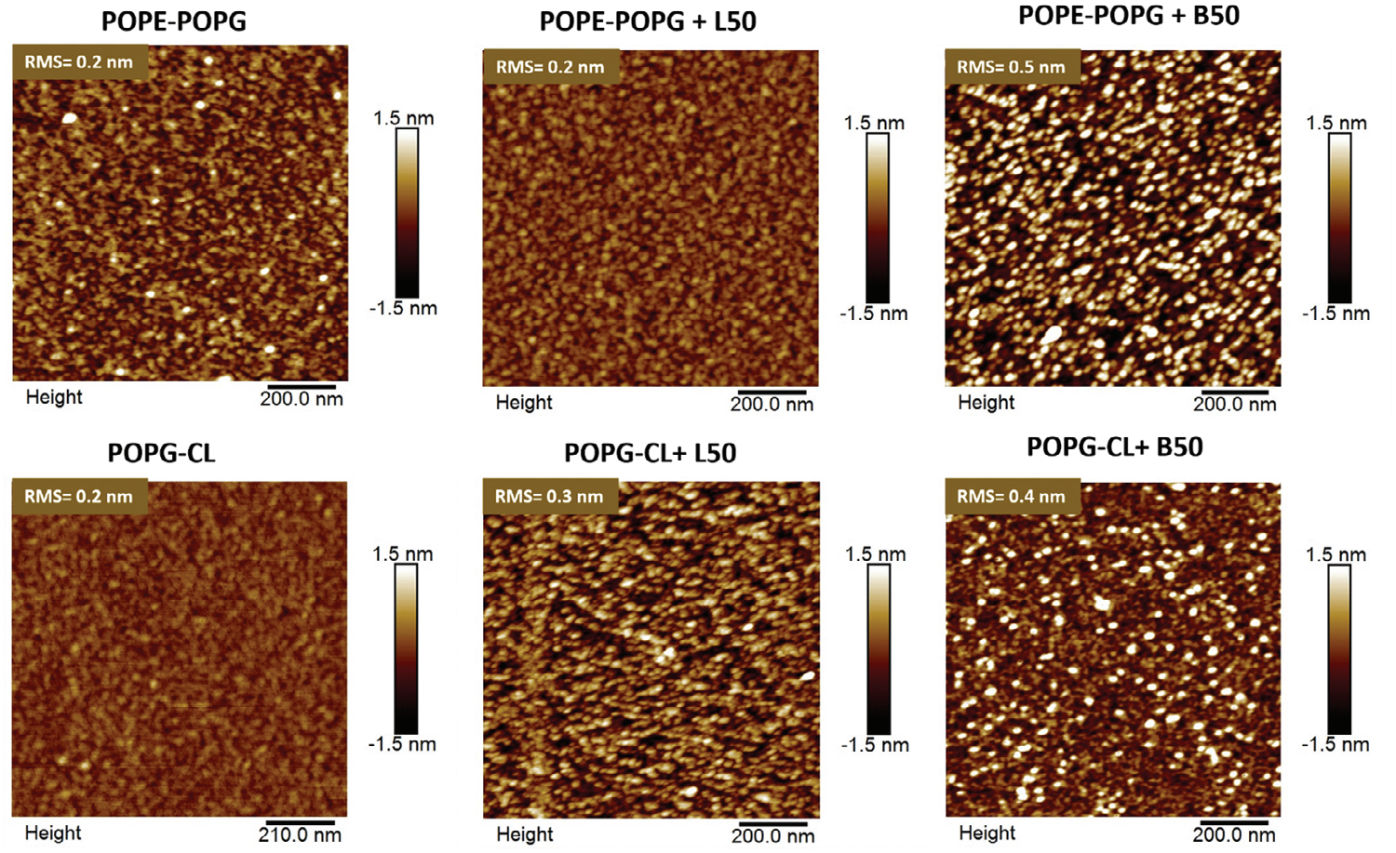
Atomic force microscopy height images recorded in air together with calculated RMS of POPE‐POPG (top) and POPG‐CL (bottom) lipid monolayers mimicking *E. coli* and *S. aureus*, respectively. Images show lipid monolayers alone (left) and monolayers incubated for 1 h with either linear L50 (center) or with bottlebrush B50 (right) APs.

AFM images of the transferred films indicated a clear difference between B50 and L50 on both monolayers (**Figures** **6**, S4 and Table S2, Supporting Information). The images show similar structures for B50 adsorbing to both lipid mixtures, with elevated structures looking somewhat denser on POPE‐POPG. This might be related to the greater density of negative charges in POPG‐CL, leading to a stronger cohesion between the lipids and B50 and thus less aggregation of the APs. The cohesive electrostatic interaction could also explain the low increase in surface pressure upon adsorption of B50 to POPG‐CL. There is also a marked difference in the adsorbed films formed by L50, which in general appear rather smooth, while the B50 films have distinct features with higher roughness. This is particularly apparent for the films scanned in water (Figure S4, Supporting Information). The dimensions of the elevated features on the membranes after treatment with B50 suggest that individual brush copolymers are visible here. This indicates that electrostatic repulsive interaction between multivalent brush copolymers prevents intermolecular polymer agglomeration on the monolayer. Such individual charged molecules could act as local defects and cause membrane distortion. In contrast, the layers after adsorption of L50 are in most cases as smooth as the bare monolayer, suggesting that the linear APs are adapting to the surface and overlapping as they do in solution. Such a homogeneous covering of the membrane surfaces may not be as disruptive as the adsorption of individual dense brushes with dense charges creating line tension.

A more quantitative assessment of the structures of the films via the root mean square (RMS) roughness and the radial distribution functions (G(r)) also supports the observation that B50 was forming rougher and more granular layers upon adsorption to lipid monolayers when compared to L50 (Table S2, Supporting Information).

Further information about the structure of Langmuir‐monolayers formed by POPG‐CL and POPE‐POPG, as well as on the perturbation caused by the addition of L50 and B50, was acquired by means of Neutron Reflectivity (NR) measurements. In NR experiment a collimated neutron beam impinges on the interface at defined angles, *θ*, and the specular reflectivity *R* is measured as a function of the momentum transfer perpendicular to the interface, *Q*_z_:

(1) $$Q_{z}=\frac{4\pi }{\lambda }\;\sin\theta$$

where λ is the neutron wavelength. The reflectivity, *R*, is defined as the ratio between the specularly reflected intensity at a given *Q*_z_ and the intensity of the incident beam. The specular reflectivity is connected to the Fourier transform of the scattering length density (SLD) profile along the direction which is normal to the interface. In turn, the SLD describes how strongly a given medium will scatter the neutrons and depends on the medium composition. Then, the analysis of NR profiles may give information of the chemical and isotopic composition and structural organization perpendicular to the interface.^[^ ^51^ ^]^ The partial SLDs and partial molecular volumes of the molecules used in the investigations are reported in Table S3 (Supporting Information).

The data analysis of NR profiles involves the inversion of the Fourier transform of the SLD profiles which is an ill‐posed problem, i.e., different (and often unphysical) sets of parameters (such as thickness of layers and roughness of the interfaces) can equally well describe the experimental reflectivity profile. To reduce this ambiguity, the same system is investigated while varying the isotopic composition, referred to as isotopic contrasts, mainly by replacing (totally or partially) some hydrogenated species with the corresponding deuterated ones. In the present study, the Langmuir‐monolayers composed by POPG‐CL or POPE‐POPG (respectively mimicking membranes of *E. coli* and *S. aureus*) in the absence and after injection of the antimicrobial polymers L50 and B50 were analyzed on two subphases, namely D_2_O and a mixture of H_2_O/D_2_O having an SLD equal to air.

Figure S7 (Supporting Information) reports the experimental data obtained from NR measurements. Due to the lack of scattering contrast of the lipid monolayers in air contrast matched water (ACMW; a mixture H_2_O/D_2_O with 0.0808 mole fraction of heavy water), this contrast is particularly sensitive to the adsorption of additional material to the interface, manifested as an increase in scattering intensity at low *Q*_z_ arising from AP adsorption as surface excess increases.^[^ ^52^, ^53^ ^]^ Visual comparison of the reflectivities at low *Q*‐values for systems in ACMW with and without polymer allows describing in a qualitative way the effect of the polymer injection following the trend:

(2) $$\begin{array}{c} \left(\mathrm{POPG}-\mathrm{CL}+B50\right)>\left(\mathrm{POPE}-\mathrm{POPG}+B50\right)>\\ \left(\mathrm{POPE}-\mathrm{POPG}+L50\right)∼\left(\mathrm{POPG}-\mathrm{CL}+L50\right) \end{array}$$

**Figure 7:**
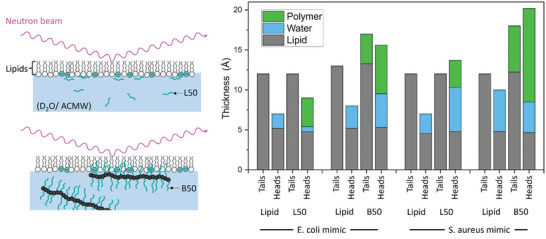
Neutron reflectometry experimental systems for investigation of the interaction between Langmuir monolayers, made of phospholipid mixtures of POPE‐POPG (8:2) or POPG‐CL (6:4) mimicking inner membrane of *E. coli* and *S. aureus*, respectively. The linear (L50) or bottlebrush (B50) APs were injected in the subphase (D_2_O/ACMW) underneath the phospholipids’ monolayer and changes induced by polymer in the tails/head of phospholipids were deduced.

Thus, the larger perturbation of both the POPG‐CL and POPE‐POPG monolayers by the bottlebrush copolymer is quite apparent. The addition of the polymers perturbs the arrangement of the pristine lipid monolayer, with insertion of the blocks in the hydrophilic as well as in the hydrophobic portion of the monolayer (**Figure** **7**). The quantitative analysis of the NR data, as described in the experimental data, suggests that while both polymers were present in the hydrophilic region of the hydrophilic heads, only the bottlebrushes were also penetrating the hydrophobic tail part of the layer.

As it can be inferred from the analysis of **Table** **1**, the parameters extracted from the NR analysis confirm the qualitative trend coming from the data reported in Figure S7 (Supporting Information). The analysis of the SLD profiles, shown in Figure S7 (Supporting Information), agrees with the results displayed in the table, in particular for what concerns the shift of the SLD distribution toward higher *z* values, due to layer thickening.

**Table 1:** Structural information of the hydrophobic and hydrophilic layers composing the Langmuir monolayer in the presence and absence of the antimicrobial polymers, as obtained from analysis of NR data. The table reports the thicknesses of the hydrophobic (τ_1_) and hydrophilic layers (τ_2_), respectively; the percentage of water in the hydrophilic layer $100\phi_{w}^{\left(2\right)}$; and the percentage of polymer in the two layers ($100\phi_{\mathrm{pol}}^{\left(1\right)}\;\mathrm{and}\;100\phi_{\mathrm{pol}}^{\left(2\right)}$). The area per lipid molecule *A*_p_values calculated for the different compositions are also reported. A detailed description of the adopted model is described in the experimental section.

System		*A*_p_/Å^2^	τ_1_/Å	$100\phi_{\mathrm{pol}}^{\left(1\right)}$	τ_2_/Å	$100\phi_{w}^{\left(2\right)}$	100$\phi_{\mathrm{pol}}^{\left(2\right)}$
POPE + POPG	–	71.6	12 ± 2	–	7 ± 2	26 ± 9	–
	L50		12 ± 2	0	9 ± 2	7 ± 10	40 ± 9
POPE + POPG	–	64.4	13 ± 2	–	8 ± 2	35 ± 9	–
	B50		17 ± 3	22 ± 9	15.6 ± 1.5	27 ± 11	39 ± 11
POPG + CL	–	98.8	12 ± 2	–	7 ± 2	35 ± 1.2	–
	L50		12 ± 2	0	13.7 ± 1.4	40 ± 15	25 ± 13
POPG + CL	–	99.1	12 ± 2	–	10 ± 3	52 ± 16	–
	B50		18 ± 3	32 ± 11	20.2 ± 1.9	19 ± 10	58 ± 11

The thicknesses and volume fractions are consistent with a picture where the graft polymer penetrate the hydrophobic and hydrophilic layers causing an increase in the thickness of these regions. In case of the hydrophobic regions, that could be realized through chain stretching or a decreased tilt angle of the lipids. In the hydrophilic region, this seems to be mainly realized through an adlayer of polymer. The thickness of the layer is approximately doubling, with the polymer taking roughly half of the volume of the hydrophilic layer. In the case of POPG + CL, also some replacement of the water by polymer is apparent. Fittings of the systems in the presence of the linear polymer L50 converge to a state where the polymer volume fraction in the hydrophobic layer is negative and has, consequently, been kept fixed to zero which is the lowest physical meaningful value. In comparison to the B50 system, the hydrophilic layers were considerably thinner, which might be attributed to the less flexible conformation of the bottlebrush polymers. In the POPE + POPG system, the linear polymer sits in the headgroup layer and replaces the water whereas the bottlebrushes are more or less below the headgroups. In case of POPG + CL, L50 is not able to penetrate the headgroups and forms a low‐density layer below, whereas the B50 forms a much thicker and denser layer below that also replaces some of the water from the headgroup layer. Thus, the higher activity of B50 could be firstly attributed to its ability to penetrate the tail‐part of the lipids, which is required to effectively disrupt lipid bilayers, and by the thicker layers formed in the head regions, which could lead to charge inversion and thus favor aggregation as seen in Figure 2. Nevertheless, the density of B50 is still below the monolayer level, as the measured increase in thickness of the headgroup layer is considerably lower than the length of the sidechains of the bottlebrushes. This is in line with the conclusions drawn from AFM images showing individual and separated structures. In terms of selectivity, it is quite obvious that the more negatively charged POPG + CL system attracts more B50 than the less negatively charged POPE + POPG system.

## 3 Conclusion

Antimicrobial copolymers of equal composition, featuring either a linear or a bottlebrush architecture, were investigated regarding their physico‐chemical properties and interaction with biological membranes. Using different analytical tools, we could identify differences in the properties of the polymers, namely an altered pK_a_ value and a difference in apparent polarity.

Our investigation revealed some unique features of bottlebrush copolymers that make them highly suitable as antimicrobial agents: 1) The confined nature of side chains within such structures stabilizes them in solution, preventing intermolecular self‐assembly and reducing their aggregation with proteins. 2) Their multivalent nature enables faster and more efficient attachment to the membrane interface and promotes aggregation between liposomes. Importantly, this is not a result of increased amphiphilic tendency or surface activity as probed in separate experiments. 3) Although the observed selectivity to bacterial strains cannot be explained by membrane models alone, as the full complexity of the cell wall with considerable glycoproteins needs to be considered, the greater negative charge density of *S. aureus*, in comparison to *E. coli* mimicking membranes, correlates with denser adlayers of the positively charged polymers. 4) Bottlebrushes bind to biological membranes in a unimolecular sense, and the perturbation of the membrane is likely caused by individual macromolecules causing local defects as opposed to ensembles of linear polymers that adapt to the surface. 5) The unique structure of bottlebrushes enables them to penetrate deeper into the bacterial membrane, interacting with the hydrophobic lipid tail region, something that linear chains of equal composition are not capable of. While there are still open questions, the present study provides insight into the profound impact of polymer topology on membrane interaction on a molecular level revealing distinct differences of the mode of action as a function of architecture.

## 4 Experimental Section

### Surface Activity

The surface activity of polymers was measured by monitoring the change in the surface tension by increasing the concentration of polymers. Microtrough and FilmwareX 4.0. settings from Kibron (Helsinki, Finland) were used to measure the surface tension. Prior to each experiment, the Kibron Dyneprobe was rinsed successively with ethanol, acetone and water and then was flamed to achieve complete wetting and placed on the sensor. The surface tension of the sensor was calibrated first in air and then at air–water interface in a Dynecup. PBS (1×) buffer (3.2 mL) was used as subphase as well as dissolution medium for polymers. Polymer stock solutions were gradually added to the subphase and left to equilibrate for about 100 s before the next polymer injection and the final surface tension was recorded and plotted against the respective final polymer concentrations (log scale) in the Dynecup with OriginPro 2022b.

### High Pressure Liquid Chromatography (HPLC)

HPLC measurements were performed on a 10A HPLC, Shimadzu system series fitted to a Pursuit XRs 5 C18 250 × 4.6 mm (100 Ǻ) reverse phase column at room temperature. 25 µL of polymer (5 mg mL^−1^) in milliQ water were injected using a Shimadzu SIL‐20A HT autosampler. UV detection was monitored at 309 nm wavelength. A gradient elution was applied with mobile phase A made of HPLC grade water + 0.04% TFA and mobile phase B made of HPLC grade acetonitrile + 0.04% TFA. The elution was performed first with 99% solvent A up to 10 min followed by gradient elution of 1–95% from 10 to 50 min and maintained at 95% solvent B until 60 min.

### Potentiometric Titration

A general‐purpose pH probe connected to a Mettler Toledo pH meter was used for potentiometric titration. The pH meter was rinsed with deionized water and calibrated with standard buffers (pH 4, pH 7, and pH 10). All solutions were prepared with milliQ water and titrations were carried out at ambient temperature (25 °C) and under gentle mixing with a magnetic stirrer. The titrate solution (3 mL) prepared at a concentration of 1 mg mL^−1^ of polymers in HCl (0.01 m) was placed in the titration cell (10 mL scintillation vial). NaOH (0.1 m) was gradually added as titrant with Eppendorf micropipettes (from 20 to 600 µL). Sufficient time (about 1 min) was necessary to reach a stable pH reading before the next addition of the base. Recorded pH values were plotted against respective cumulative volumes of the titrant to obtain the titration curve and the first derivatives were used to determine the equivalence points and corresponding pK_a_.

### Liposomes Preparation

Phospholipids mixtures were used to prepare liposomes mimicking membranes of *S. aureus*, *E. coli*, and red blood cells. The composition of *S. aureus* like membrane was made of 2‐Oleoyl‐1‐palmitoyl‐sn‐glycero‐3‐phospho‐rac‐(1‐glycerol) sodium salt (6 mg, 7.8 µmol) and 18:1 Cardiolipin (4 mg, 2.7 µmol). For *E. coli*‐liposomes, 2‐Oleoyl‐1‐palmitoyl‐sn‐glycero‐3‐phosphoethanolamine (8 mg, 11.1 µmol) and 2‐Oleoyl‐1‐palmitoyl‐sn‐glycero‐3‐phospho‐rac‐(1‐glycerol) sodium salt (2 mg, 2.6 µmol). And for liposomes mimicking red blood cells a mixture of 1,2‐dioleoyl‐sn‐glycero‐3‐phosphocholine (9 mg, 11.4 µmol) and 1,2‐dioleoyl‐sn‐glycero‐3‐phospho‐L‐serine (sodium salt) (1 mg, 1.23 µmol). The respective lipid mixtures were dissolved in of CHCl_3_ (1 mL) in a 25 mL round bottom flask. A thin film lipid was formed by drying the organic solvent on the Rotavap under vacuum. The thin film was subsequently hydrated with phosphate buffer saline (1 mL PBS 1×) and stirred for 1 h at room temperature. The suspension was then extruded 15 times using polycarbonate membranes of 400 and 100 nm successively. The liposomes were stored at 4 °C in a fridge and used within two weeks.

### Dynamic Light Scattering (DLS)

DLS measurements of polymers in milliQ and Müller Hinton Broth (MHB) were performed on a Zetasizer Ultra from Malvern Panalytical, (United Kingdom) at a measurement angle of 173° and temperature of 25 °C. Polymer solutions (1mg mL^−1^) dispersed in milliQ or in MHB media were placed in a disposable 4 mL polystyrene cuvette. The hydrodynamic radius of polymers was derived from Z‐average diffusion for 3 measurements points in a time series. To check the interaction of the three types of vesicle models with the polymers, a spectroLight 610 Xtal‐concept (Germany) with a microplate reader was also used to investigate the polymer–vesicles interactions. For this, liposomes suspensions (0.5 mg mL^−1^) were incubated for at least 10 min with polymer (512 µg mL^−1^) solution in PBS at 25 °C (polymer‐liposome volume ratio of 1:100). 0.8 µL of liposomes alone and mixture of liposomes with L50 and B50 were spotted on the plate reader for DLS measurements were performed after drop search.

### Cryogenic Electron Microscopy (Cryo‐EM)

Liposome suspensions (0.5 mg mL^−1^) mimicking *S. aureus*, *E. coli*, and RBC membranes were used for cryo‐EM. The polymers, namely L50 and B50, were dissolved in PBS (512 µg mL^−1^) and a 1/100 volume ratio of polymer/liposome was prepared before grid preparation. 5 µL of liposome suspension, with or without polymers, was applied on freshly glow discharged Cu 400 mesh Carbon support grids (Quantifoil) and incubated for 5 min at 10 °C and at 90% humidity. Afterward, grids were blotted for 5 s and plunged frozen in liquid ethane by using an EM GP2 (Leica) automated plunge freezing device.

Cryo‐EM images were collected under low‐dose conditions on a Talos F200C (Thermo Fisher Scientific) transmission electron microscope operated at 200 kV equipped with a 4k × 4k Ceta 16M CMOS camera. Micrographs were recorded by using the Velox software (Thermo Fisher Scientific) at a magnification of 13.500× resulting in a pixel size of 10.68 Å pix^−1^ or at 57.000× magnification resulting in a pixel size of 2.53 Å pix^−1^.

Liposome dimensions (length and width) were measured by using the image processing package Fiji.^[^ ^54^ ^]^ The aspect ratio was then calculated by dividing the length to the width of isolated vesicles (*n* > 150) and graphs were plotted using OriginPro 2022b software.

### Quartz Crystal Microbalance with Dissipation (QCM‐D)

A four‐chamber Q‐Sense E4 system (Biolin Scientific) was used for QCM‐D measurements on silicon oxide sensors crystals (QSX 303). Before experiments, the crystals were successively cleaned in acetone, methanol, milliQ water, then air‐dried, and finally, plasma cleaned. The cleaned crystals were directly placed in the QCM‐D chambers and resonance frequency (*∆f*) and dissipation (*∆D*) shift data were collected from 4 overtones (3rd, 5th, 7th, and 9th). Degassed phosphate buffer saline (PBS) buffer was injected up to when a constant baseline of *∆f* and *∆D* was obtained. Then liposomes solutions (0.5 mg mL^−1^) made of DOPC‐DOPS (9:1 mass ratio) were injected to form the lipid bilayer on the SiO_2_ crystals. The flow of liposomes was maintained until stable signals of *∆f* and *∆D* were obtained. Excess of lipids were then rinsed with PBS before injection of polymer solutions (in PBS) at a concentration of 300 µg mL^−1^. Finally, the crystals were rinsed by a consecutive flow of PBS and milliQ water. Throughout the experiment, the flow rate of 100 µL min^−1^ was maintained using an IPC peristaltic pump. To prevent the formation of air bubbles in the tubing system, the flow rate was interrupted shortly each time the solutions were changed. Recorded values from the 5th overtone were used to determine the mass of polymers adsorbed on the supported lipid layers using the following modified Sauerbrey equation (Equation (2)):

(3) $$\frac{m}{A}=-\frac{C}{n}\left(\Delta f+\frac{f_{c}.\Delta D}{2}\right)$$

where *f*_c_ represents the resonance frequency of the crystal (*f*_c_ = 4.95 MHz), *C* for the crystal constant (*C *= 17.7 ng cm^−2^); *n* is the overtone number (*n* = 5), and *m*/*A* the adsorbed mass normalized against the apparent area. The molecular weights obtained by aqueous SEC were incorporated in Equation (3) for the chain density calculations.

The time‐resolved adsorption of the polymers was approximated with a simple exponential decay function:

(4) $$\frac{m}{A}\;\left(t\right)=\frac{m_{\mathrm{eq}}}{A}\;-\frac{\Delta m}{A}\exp\left(-\frac{t-t_{0}}{\tau }\right)$$

with *m*/*A* (*t*) at any time *t*, *m*_eq_/*A* as adsorbed mass in equilibrium, Δ*m*/*A* as adsorbed mass difference from initial to equilibrated state, Δ*m* = *m*_eq_–*m*_0_.

### Langmuir Monolayer

A Kibron (Helsinki, Finland) Microtrough and the FilmwareX 4.0. were used for preparation of the Langmuir monolayer. The G2 trough inset (350 mm length and 80 mm width) and the movable barriers were cleaned with chloroform and ethanol and then rinsed with milliQ water. The probe was also rinsed with ethanol, acetone and water and by glowing it with a gas torch. Calibration against air was done before each experiment. 200 mL of PBS were added on the trough as subphase. Prior to compression, lipid solutions in chloroform (1 mg mL^−1^, 25 µL) of either POPE‐POPG (8:2 mass ratio) or POPG‐CL (6:4 mass ratio) were spread on the subphase. Chloroform was allowed to evaporate for 10 min. The barriers were closed at a constant rate of 10 mm min^−1^ up to the targeted pressure of 20 mN m^−1^. After the targeted pressure was reached, the barriers were stopped, and the layer left to relax for 30 min. The final surface pressure at steady state was about 19 mN m^−1^. 2 mL of polymer solution in PBS (40 µg mL^−1^) were injected underneath the barriers. Prior to polymer addition, an equivalent volume of subphase was removed. The surface pressure was monitored for 1 h. Thereafter, the monolayers were transferred to pieces of silicon wafers using the Langmuir–Schäfer technique. Samples for measurements in air were dried under a nitrogen stream whereas samples for measurements in water were stored in MilliQ water.

### Atomic Force Microscopy (AFM)

AFM measurements were carried out on a MultiMode 8 (Bruker Corp., Billercia, USA). In air, in tapping mode with Bruker RTESPW 150 Tips was used. In liquid, scan assist mode was used together with scan assist tips. All images were flattened by subtracting the median of the differences between scanning lines. RMS roughness and radial distribution functions (G(r)) were calculated with the Gwyddion as quantitative metrics of the granularity of the surfaces.^[^ ^55^ ^]^

### Neutron Reflectivity (NR)

The interaction of the two polymers and Langmuir monolayers were further investigated by NR after injection of L50 and B50 beneath the hydrogenous monolayers made of either POPE‐POPG (8:2 mass ratio) or POPE‐CL (6:4 mass ratio) lipid mixtures. To better explore structural changes, two buffers with different contrasts were prepared by dissolving PBS tablets separately in D_2_O and air contrast matched water (ACMW, a mixture H_2_O/D_2_O with 0.0808 mole fraction of heavy water). To form the monolayer, chloroform solutions of the lipids (1 mg mL^−1^, 60 µL) were spread on a 500 mL subphase trough and the organic solvent was allowed to evaporate before closing the trough chamber and left to equilibrate at 37 °C. The surface area was then compressed to the desired surface pressure (20 mN m^−1^) and barriers were stopped and the monolayer left to equilibrate for 30 min. Prior to injection of the polymers, reflectivity curves of the monolayers were collected using the INTER reflectometer at the ISIS Neutron and Muon Source. A wavelength band of 1.5–17 Å was utilized across different angles (*θ* = 0.8° and *θ* = 2.3°), covering an effective *Q*_z_ range of 0.0103–0.33 Å^−1^, utilizing a footprint on the sample parallel to the beam direction of 100 mm, perpendicular to the beam direction of 50 mm, where *dQ*_z_/*Q*_z_ = 5.5%. Raw data were collected using a wavelength shifting fiber linear detector and reduced using Mantid v6.6.^[^ ^56^ ^]^ Briefly, for each angle, spectra collected on each pixel over the specular beam were summed up and normalized for incident integrated flux using a monitor positioned after collimation and before the sample. Backgrounds were subtracted from each individual angle by summing over the detector away from the specular signal and then these backgrounds were subtracted from the specular data. Reflectivity was obtained by normalizing by a flux‐normalized direct beam measurement. Finally, individual angles were stitched and re‐binned to the instrumental resolution (*dQ*_z_/*Q*_z_ = 5.5%). After measurement of the pristine monolayers, polymer solutions prepared in the same buffer contrast as the subphase were injected in the subphase underneath the monolayer. The surface pressure was monitored while kinetic NR measurements were carried out. After 1 h of equilibration, structural NR measurements were performed on the equilibrium structure formed between the monolayers and polymers.

NR data analysis has been carried out modeling the systems as two homogenous slabs, composed of the hydrophilic (hydrated) heads and the hydrophobic tails, respectively, the latter pointing toward the upper phase (air). Due to the high areal density of lipids and the large molecular volume of the hydrophobic tails, it was assumed that the hydrophobic layer does not contain solvent. The SLDs can be used to determine the fraction of polymer and water that has penetrated the two regions of the monolayer. To reduce the number of fitting parameters, and to ensure that the models are physically realistic, the models were based on the quantity of phospholipids used to prepare each monolayer, as well as the area available for the monolayer itself, giving the area per molecule *A*_p_. Through trivial geometric considerations, it is possible to correlate *A*_p_ with the thickness of the hydrophobic and hydrophilic layers, τ_1_ and τ_2_, as well as with the amphiphile volume fraction present in each layer, ϕ_tail_ and ϕ_head_:

(5) $$A_{p}=\frac{v_{\mathrm{tail}}}{\tau_{1}\cdot \;\phi_{\mathrm{tail}}\;}=\frac{v_{\mathrm{head}}}{\tau_{2}\cdot \;\phi_{\mathrm{head}}\;}\;$$

where *v*_tail_ and *v*_head_ are the molecular volumes of the tails and heads of the phospholipids. The previous equations reduce the number of parameters, since the thickness of each slab is linked to its SLD via the volume fractions and partial SLDs of its constituents:

(6) $$\begin{array}{ccc} \mathrm{SL}D_{\mathrm{slab}} & = & {\mathrm{SLD}}_{\mathrm{tail}/\mathrm{head}}*\cdot \;\phi_{\mathrm{tail}/\mathrm{head}}+{\mathrm{SLD}}_{\mathrm{polymer}}\cdot \;\phi_{\mathrm{polymer}}+\\ & & {\mathrm{SLD}}_{\mathrm{solvent}}\cdot \;\phi_{\mathrm{solvent}} \end{array}$$

Furthermore, the roughness of the interfaces has been kept at a fixed value coming from the presence of capillary waves^[^ ^57^ ^]^ and from the knowledge of the interfacial pressure (20 mN m^−1^). According to the square‐root relation between surface tension and surface roughness, the roughness/interfacial width of all interfaces was fixed at 3.3 Å.^[^ ^58^ ^]^ The partial SLDs and molecular volumes of the lipids and the SLD of the polymers were calculated using the NIST calculator, factoring in the stochiometric composition of the lipid mixtures. Further layer representing the hydrated polymer between the water subphase and the polar region of the lipids has also been added but that analysis shows that such a third layer could not be distinguished. Analysis of NR data has been carried out with a least square self‐written C++ routine using the Abeles approach to define the slabs, with the constraints between parameters as described previously, through simultaneous fittings of the corresponding systems in the two different contrasts D_2_O (SLD = 6.34 × 10^−6^ Å^−2^) and ACMW (SLD = 0 Å^−2^).

## Conflict of Interest

The authors declare no conflict of interest.

## Supporting information



Supporting Information

## Data Availability

The data that support the findings of this study are available from the corresponding author upon reasonable request.
