# Supplementary material for: Antimicrobial Polymers at the Membrane Interface: Impact of Macromolecular Architecture
Source: Small. 2024 Dec 30;21(8):2406534. doi: 10.1002/smll.202406534 (PMC11855246; doi:10.1002/smll.202406534)
Supplement: Supplementary file 1 — Supporting Information [file SMLL-21-2406534-s001.docx]

Supporting Information

**Antimicrobial Polymers at the Membrane Interface: Impact of Macromolecular Architecture**

Alain M. Bapolisi, Anne-Catherine Lehnen, Rainhard Machatschek, Gaetano Mangiapia, Eric Mark, Jean-Francois Moulin, Petra Wendler, Stephen C. L. Hall, Matthias Hartlieb*

A.M. Bapolisi, A-C. Lehnen, M. Hartlieb

Institute of Chemistry, University of Potsdam, Karl-Liebknecht-Straße 24-25, 14476 Potsdam, Germany
E-mail: mhartlieb@uni-potsdam.de

R. Machatschek

Institute of Active Polymers, Helmholtz-Zentrum Hereon, Kantstraße 55, 14513 Teltow, Germany

G. Mangiapia, J-F. Moulin

German Engineering Materials Science Centre (GEMS) am Heinz Maier-Leibnitz Zentrum (MLZ), Helmholtz-Zentrum Hereon, Lichtenbergstr. 1, 85748 Garching bei München, Germany

E. Mark, P. Wendler

Institute of Biochemistry and Biology, Department of Biochemistry, University of Potsdam, Karl-Liebknecht Strasse 24-25, 14476 Potsdam, Germany

A-C. Lehnen, M. Hartlieb

Fraunhofer Institute for Applied Polymer Research (IAP), Geiselbergstraße 69, 14476 Potsdam, Germany

S.C.L. Hall

ISIS Neutron and Muon Source, Rutherford Appleton Laboratory, Didcot OX11 0QX, UK

**Table S1 :** Summary of aqueous characterisations of linear L50 and bottlebrush B50 copolymers

|  | **Mean size (nm)** | **Zeta potential (mV)** | **pK_a_** |
| --- | --- | --- | --- |
| B50 | 4 | 54 ± 6 | 7.9 |
| B50+MHB | 11 | - | - |
| L50 | 169 | 53 ± 9 | 8.8 |
| L50+MHB | 484 | - | - |


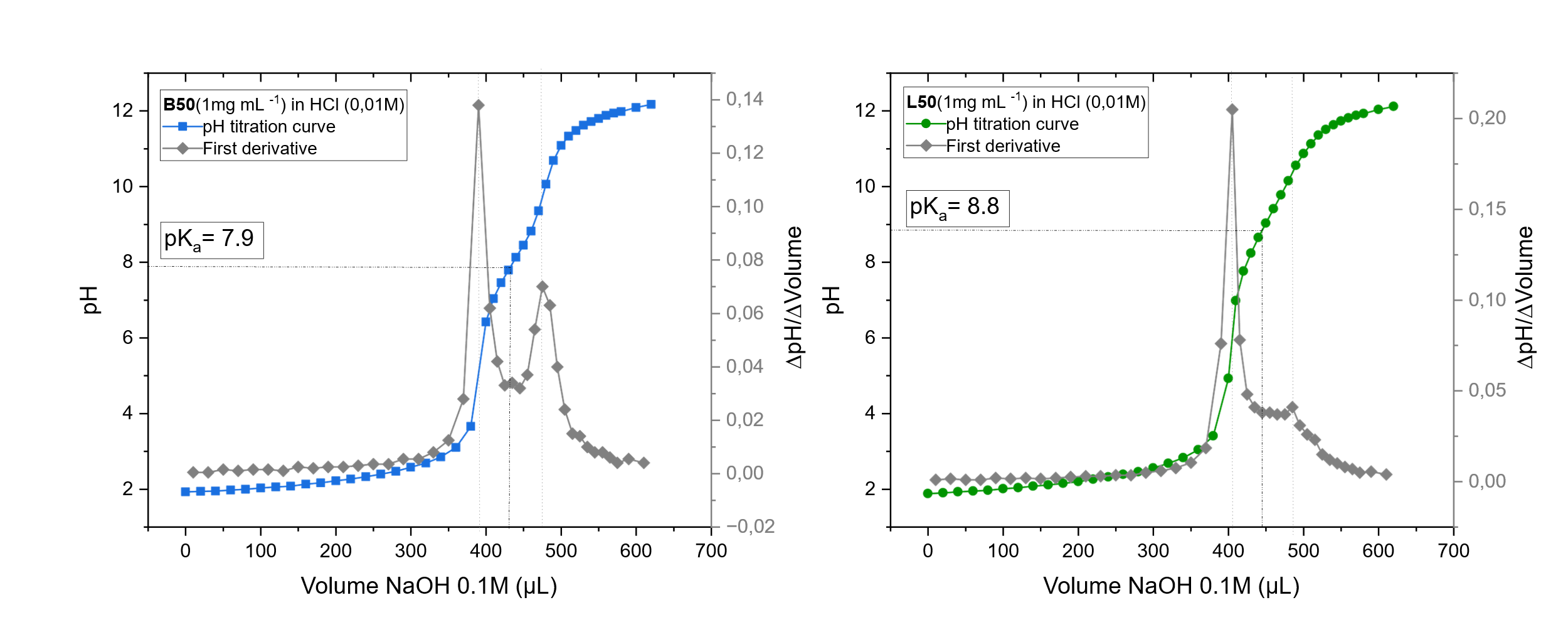


**Figure S1 :** Potentiometric titration curves (in blue for B50 and in green for L50) and their first derivatives (gray lines). The polymers were dissolved in HCl (0.01 M), at a concentration of 1 mg mL^-1^) and titrated with NaOH (0.1 M). Recorded pH values were plotted against respective cumulative volumes of the titrant to obtain the titration curve and the first derivatives were used to determine the equivalence points and corresponding pK_a_.


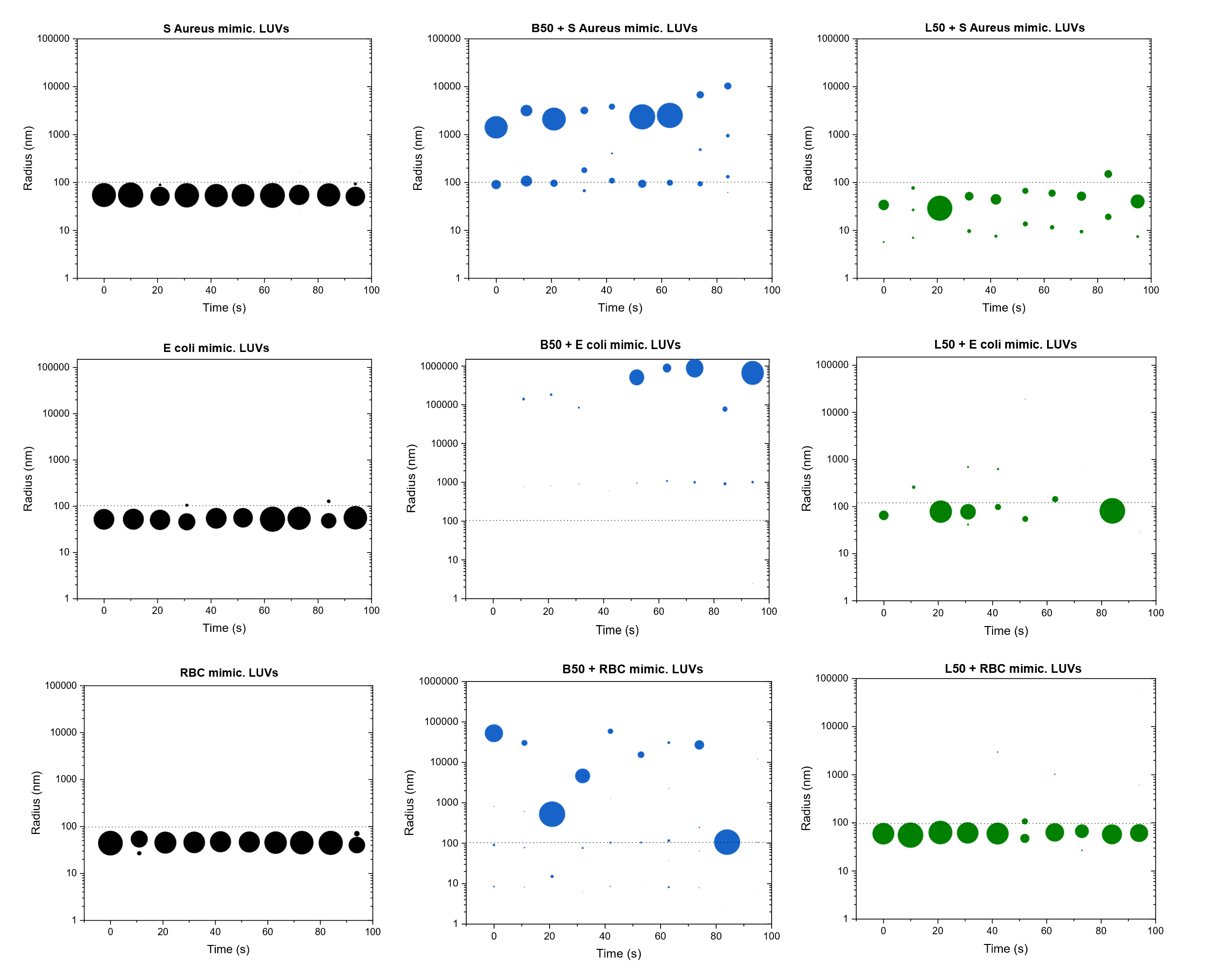


**Figure S2:** DLS radius distribution of liposomes membranes models alone (black, left), and liposomes incubated with bottlebrush polymers B50 (blue, center) and with linear L50 (green, right)) at the concentration of 512 µg mL^-1^. A spectroLight 610 was utilized to study the liposomes aggregation and data are presented as normalized amplitudes of the recorded radius. Radius values for DLS represent the hydrodynamic mean size distribution by intensity of the LUVs and the circles represent the normalized amplitudes of respective distributions recorded at each measurement in function of time (number of measurements =10).


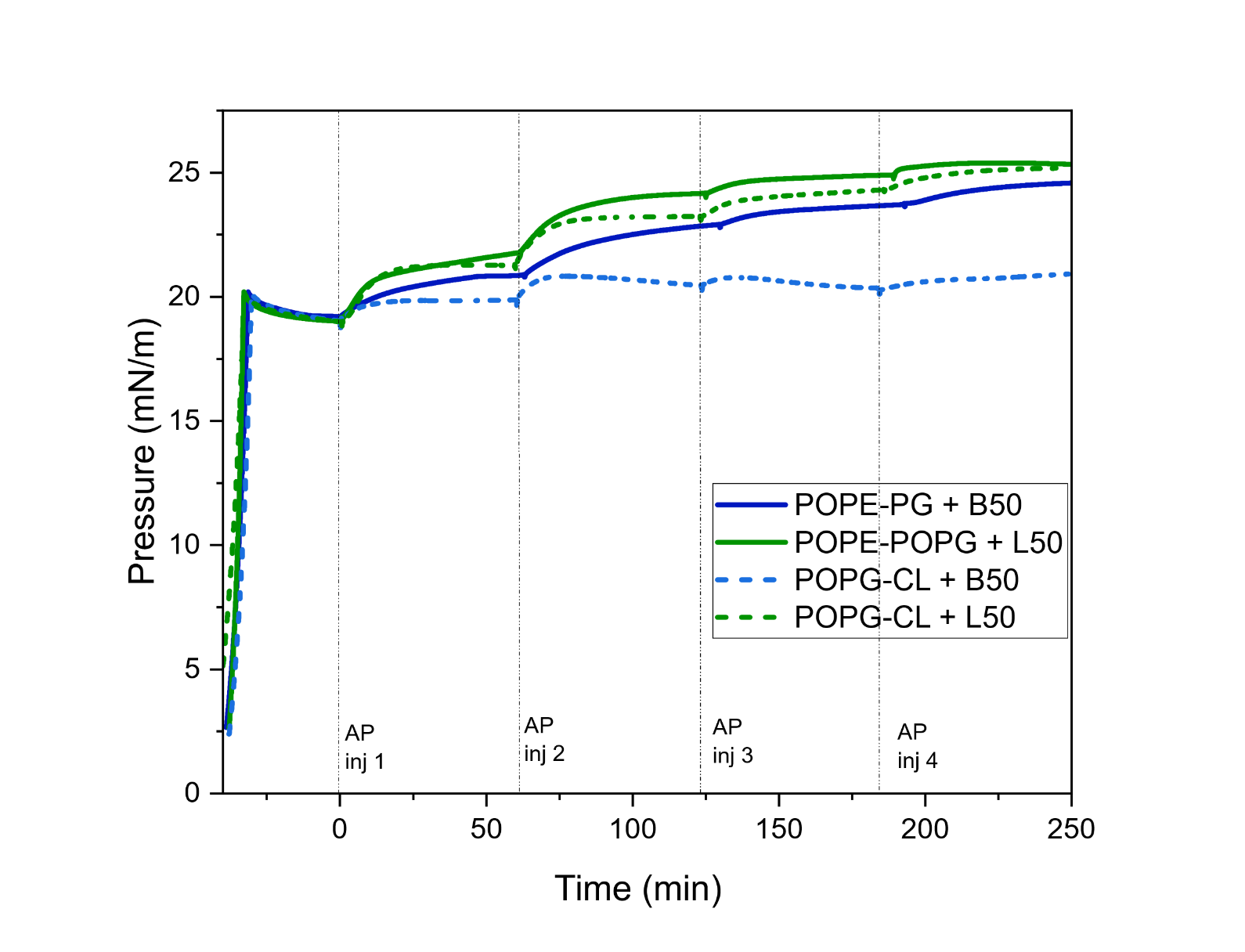


**Figure S3** : Surface pressure changes over time induced by four repetitive injection of polymers B50 (in blue) and L50 (in green) on the trough to reach final polymer concentration of 0.4 µg mL^-1^) under Langmuir monolayers earlier compressed and stabilized at 19 mN m^-1^. The monolayers were made of POPG-CL (6:4 mass ratio) (small dash lines) and POPE-POPG (8:2 mass ratio) (plain lines) lipids (1 mg mL^-1^).

| 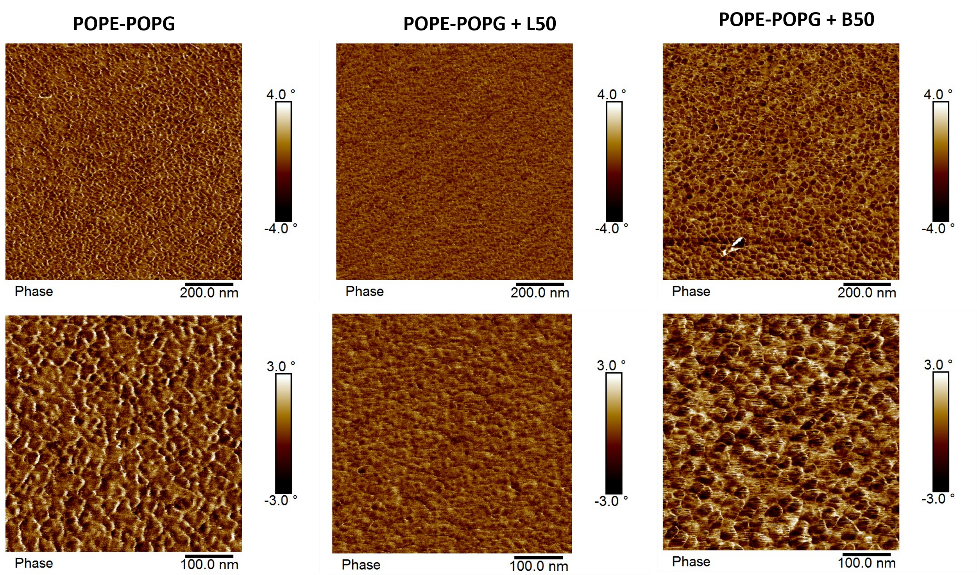 | 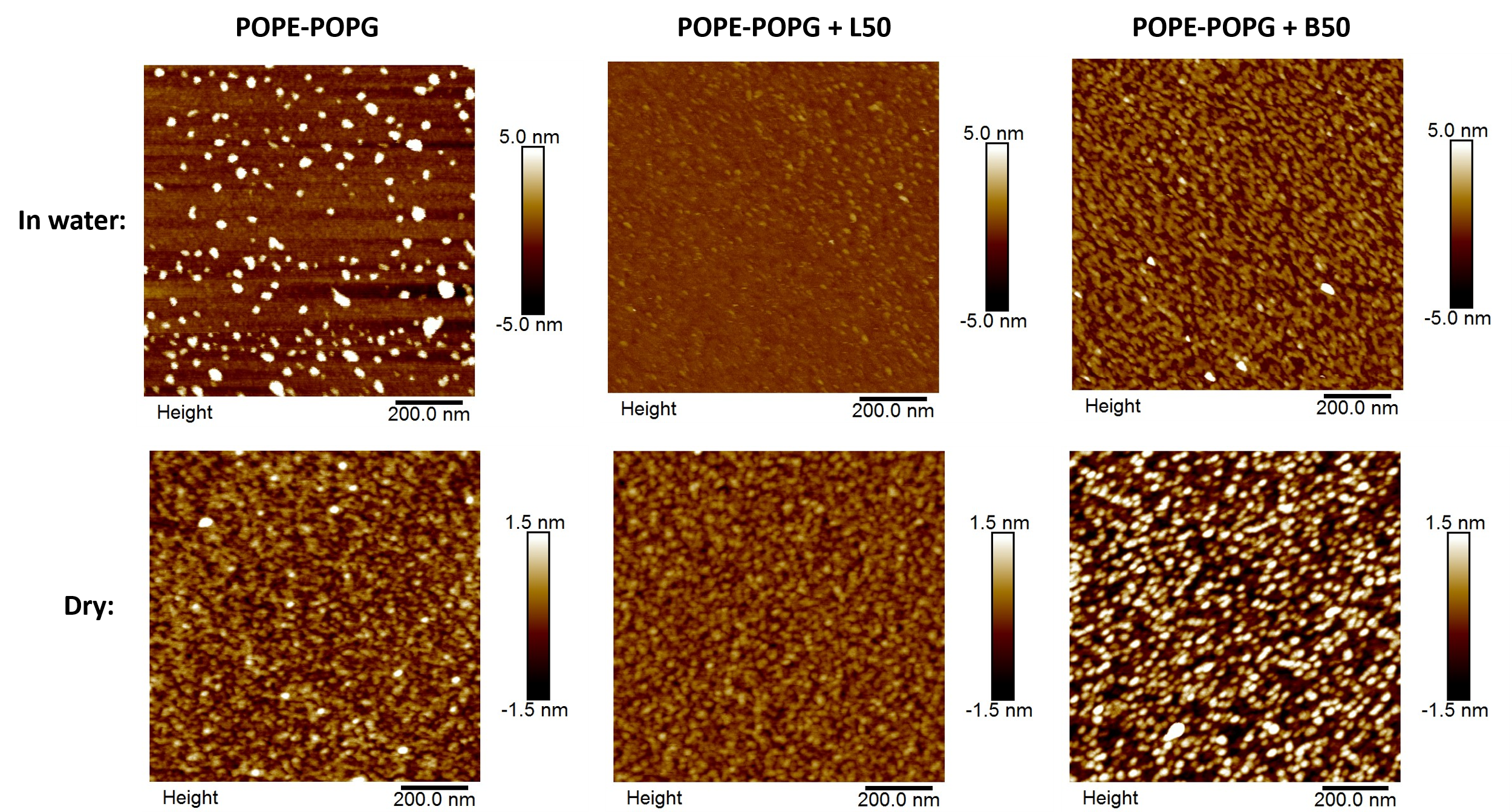 |
| --- | --- |
| 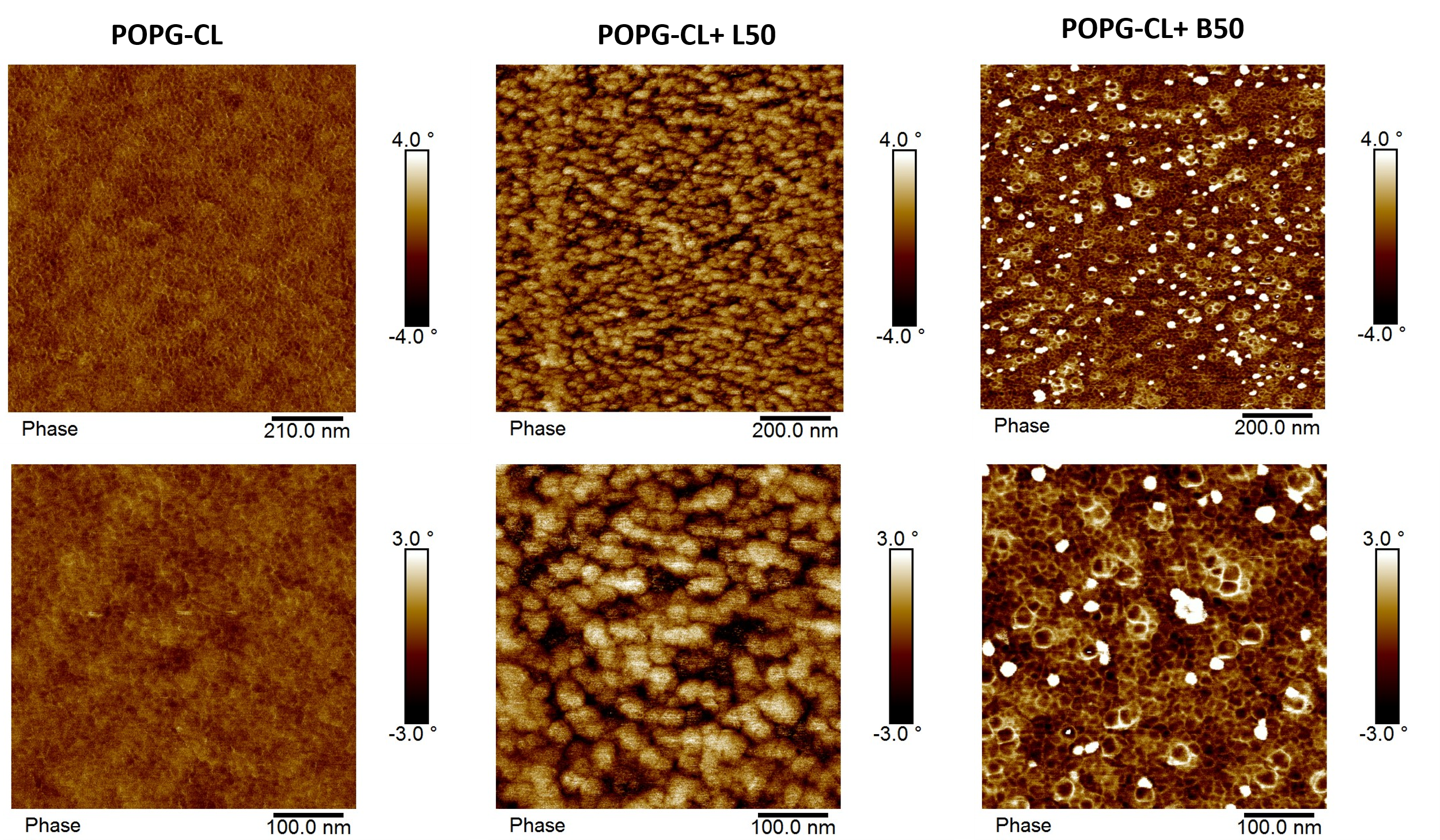 | 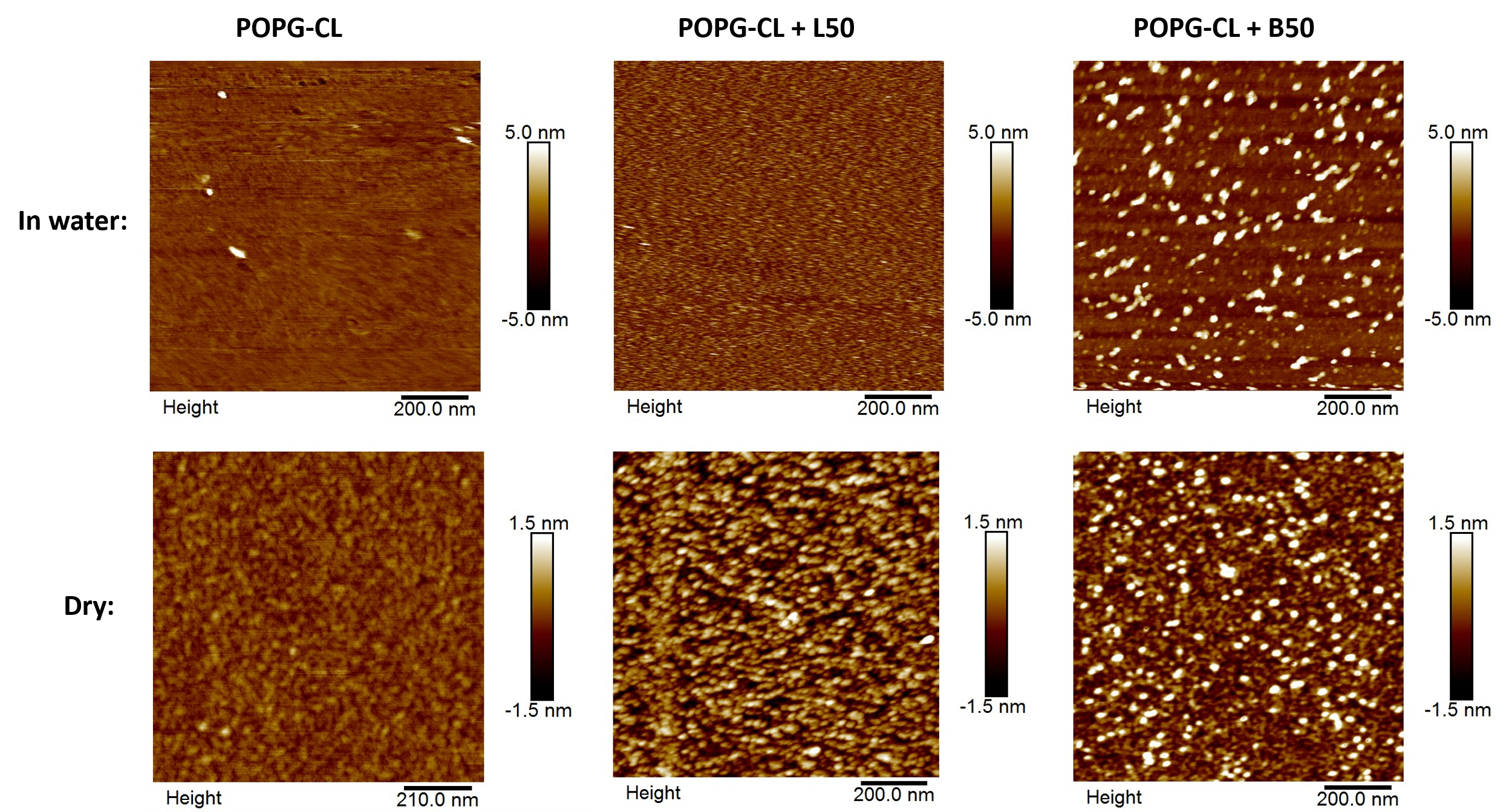 |

**Figure S4 :** Representative AFM images of POPE-POPG (top) and POPG-CL (bottom) lipid monolayers mimicking E. coli and S. aureus, respectively, from films stored in water (wet) or dry films of lipid monolayers alone (left), monolayers incubated for one hour with linear L50 (centre) and with bottlebrush B50 (right). Presented phase images were recorded on samples in dry conditions.


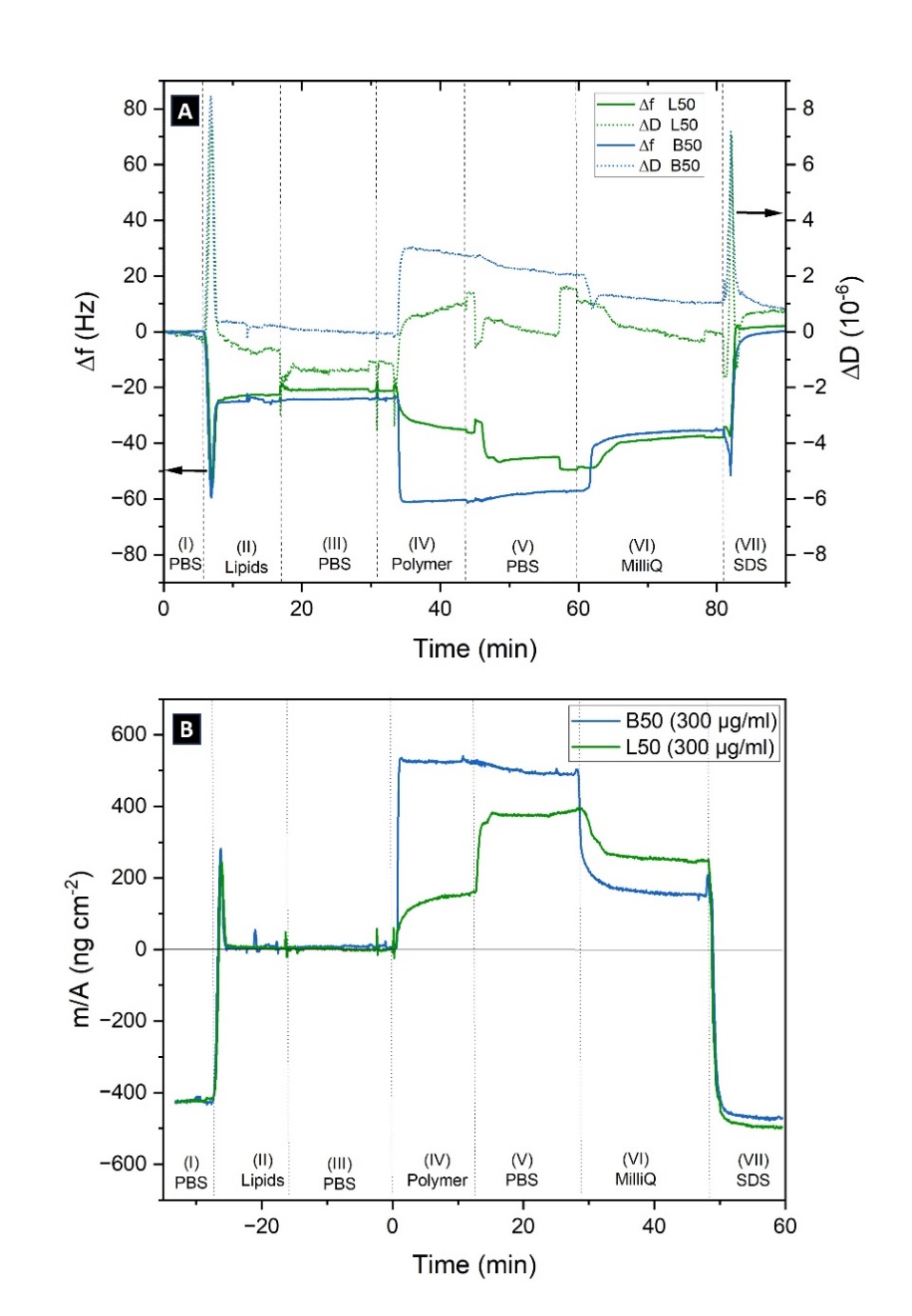


**Figure S5 :** Recorded raw QCM-D data (5^th^ overtone) (**A**); solid lines representing the frequency ∆f and dash lines the dissipation ∆D and varying at different phases: [I] calibrated baseline with PBS (1X) at pH 7.4, [II] injection of DOPC-DOPS vesicles (0.5 mg mL^-1^) to form typical supported lipid bilayer (SLB), [III] rinsing of extra lipids with PBS, no mass desorption confirming formation of stable SLB, [IV] injection of polymers L50 and B50 (150 µg mL^-1^) resulting in adsorption of polymer on formed SLB, [V] rinsing with PBS indicating structural rearrangement of polymers, [VI] and then rinsing with milliQ water resulting in partial desorption of polymers, [VII] and final wash with Sodium dodecyl sulfate (SDS 2%) to completely clear the crysrals. The kinetics of mass adsorption/desorption of polymers extracted from phase raw data is presented as mass per area (m/a) (**B**). The time of injection of polymer is considered as initial time (Time= 0 min) for time, frequency and dissipation normalization. The data were fitted with Sauerbrey equation.

| 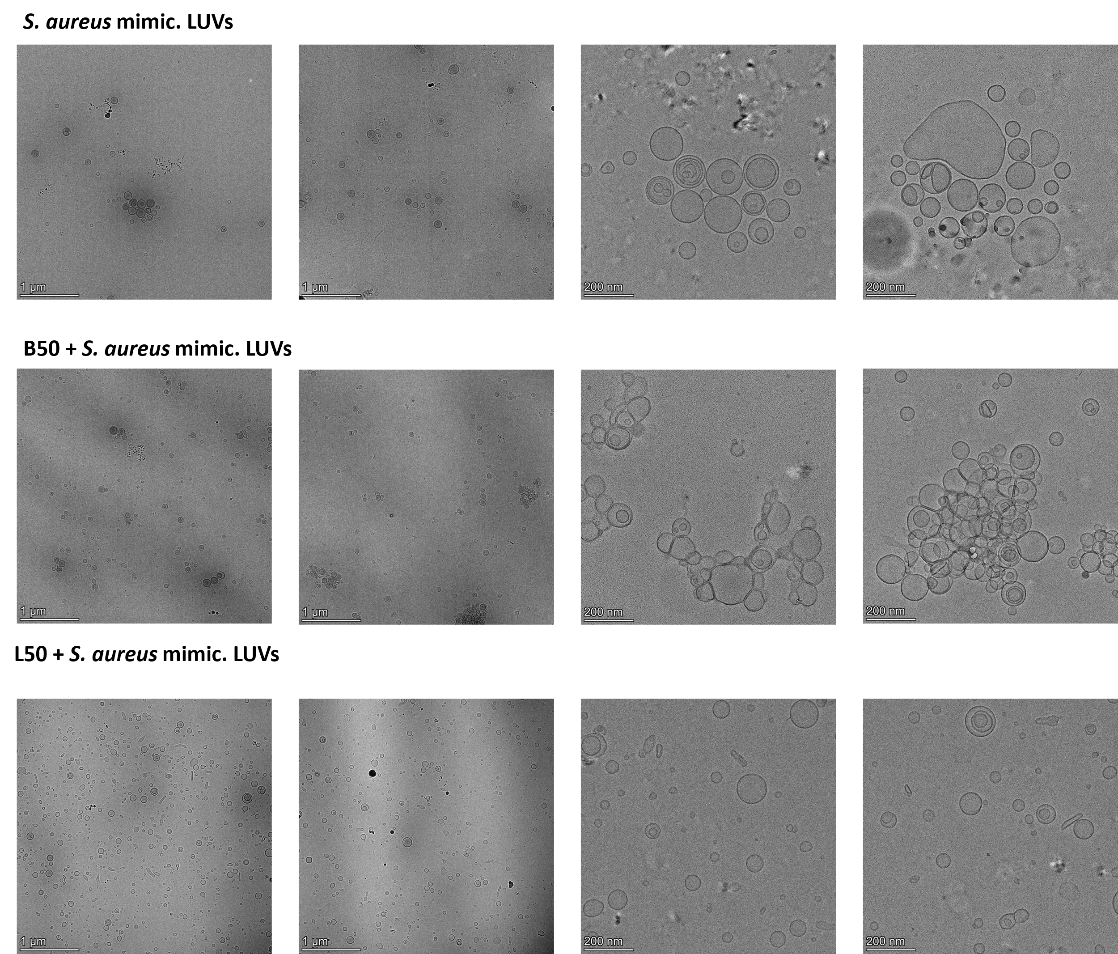 | **Figure S6:** Representative Cryo-EM images of large unilamellar vesicles (LUVs) mimicking *S. aureu*s, *E. coli* and red blood cells (RBC). For each membrane model, images of LUVs (0.5 mg mL^-1^) alone and incubated with APs (512 µg mL^-1^) of bottlebrush topology (B50) and linear copolymers (L50 on the right) were compared. |
| --- | --- |
| 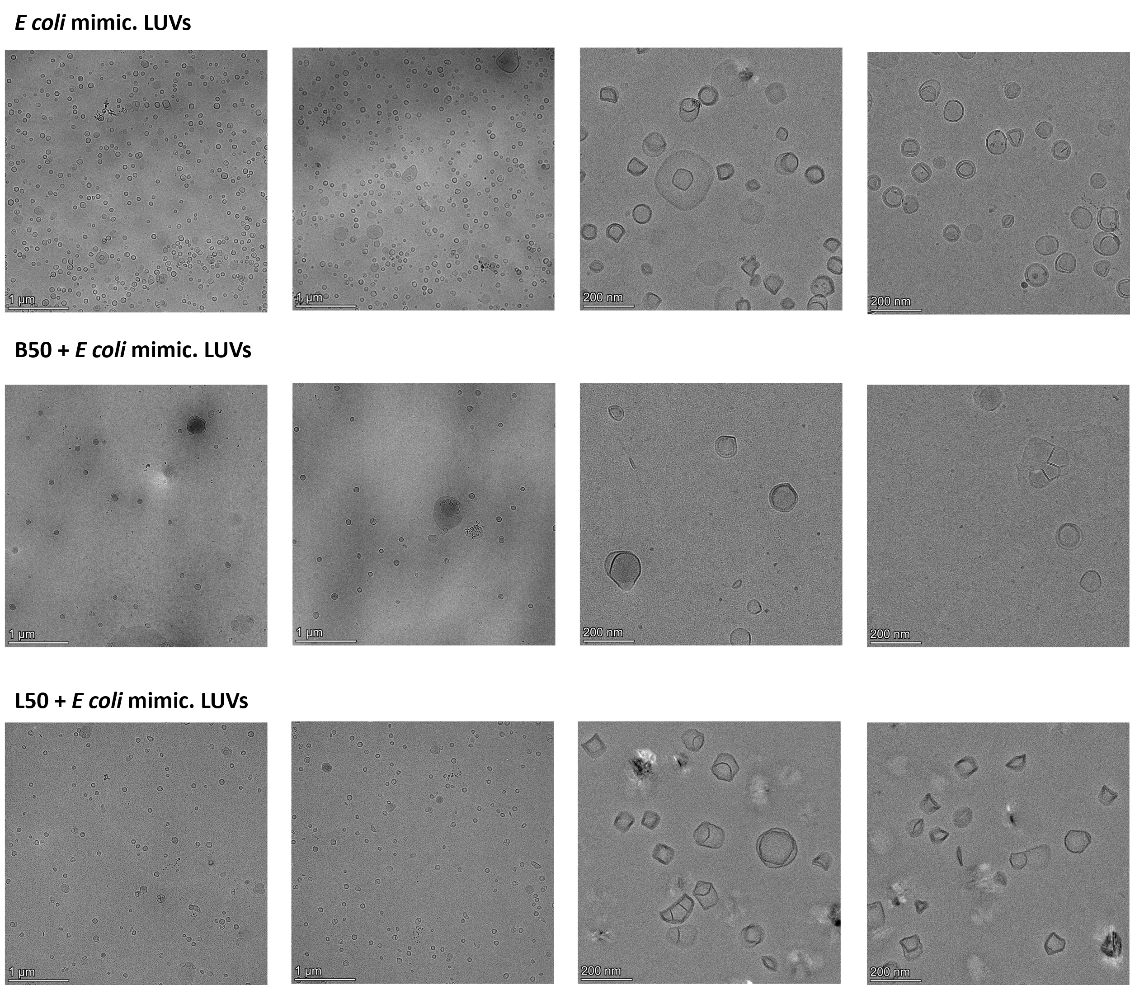 |  |
| 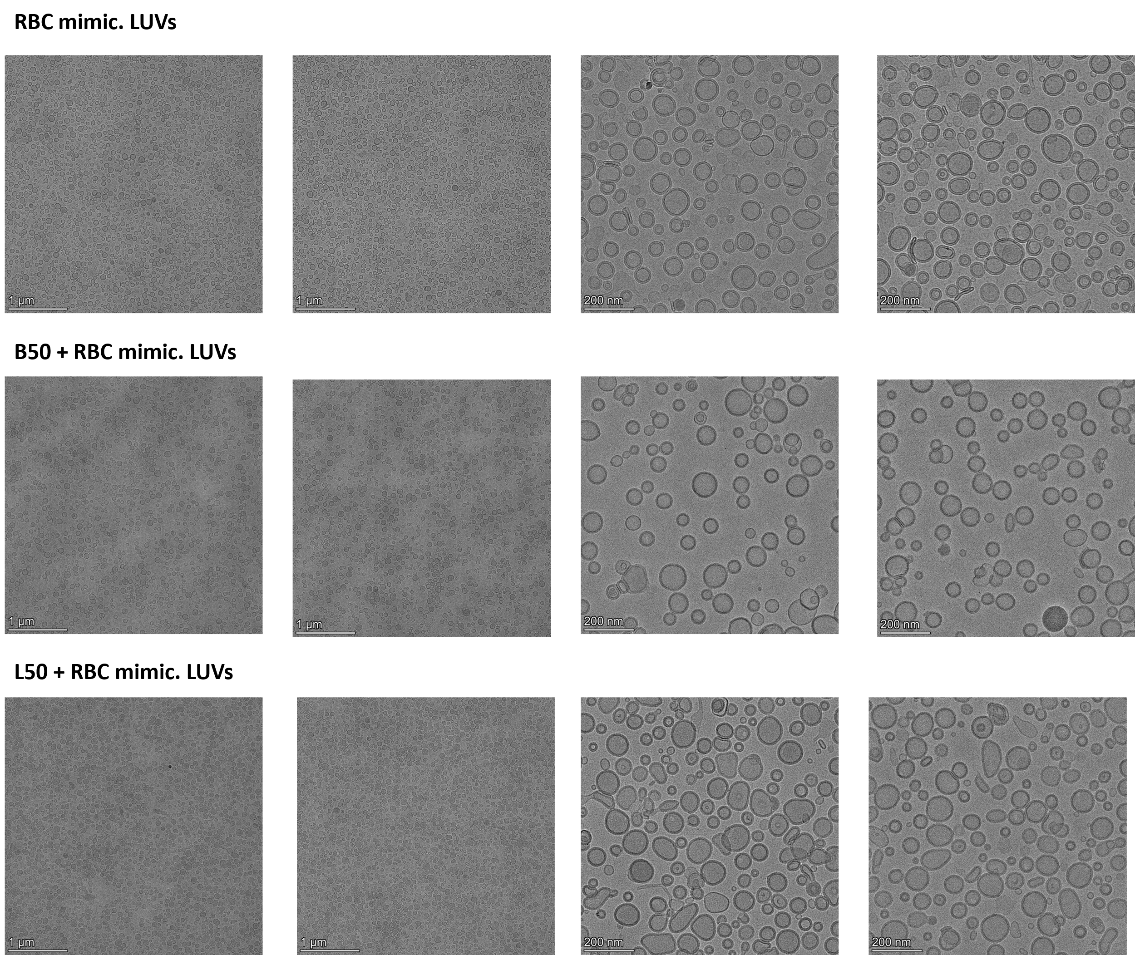 |  |

**Table S2:** Summary of derived statistical values from AFM images of dry and wet samples

| **Lipid composition** | **Polymer** | **RMS roughness (nm)** | | **Lateral Feature Size** | | **Typical Feature Height** | | **Image Quality** | |
| --- | --- | --- | --- | --- | --- | --- | --- | --- | --- |
|  |  | Dry | Wet | Dry | Wet | Dry | Wet | Dry | Wet |
| POPG-CL | - | 0.15 | 0.3 | 30 | none | <1 | <1 | Good | Bad |
| POPG-CL | L50 | 0.33 | 0.6 | 26 | 15+35 | 2 | 2-3 | Good | Average |
| POPG-CL | B50 | 0.4 | 1.0 | 30+45 | 25 | 2,5 | 6 | Good | Good |
| POPE-POPG | - | 0.2 | 1.0 | 35 | 30 | <2 | 6 | Good | Dubious |
| POPE-POPG | L50 | 0.2 | 0.2 | 20 | 20 | <1 | 1 | Good | Dubious |
| POPE-POPG | B50 | 0.5 | 0.8 | 30+45 | 20 | 2-2,5 | 2-2.5 | Good | Good |

**Table S3:** SLD and molecular volume of the lipid mixtures used for the analysis of NR data. For the antimicrobial copolymers, because of the scarce contrast existing between the hydrophilic and hydrophobic blocks, the global averaged SLD value has been used.

| **Component** | **10^6^ SLD (Å^–2^)** | | **Molecular volume (Å^3^)** |
| --- | --- | --- | --- |
| POPE-  POPG (8:2) | Tail | -0.312 | 852.6 |
|  | Polar group | 1.876 | 346.64 |
| POPG-CL (6:4) | Tail | -0.2055 | 1209.72 |
|  | Polar group | 2.136 | 464.08 |
| L50/B50 | 0.817 | |  |


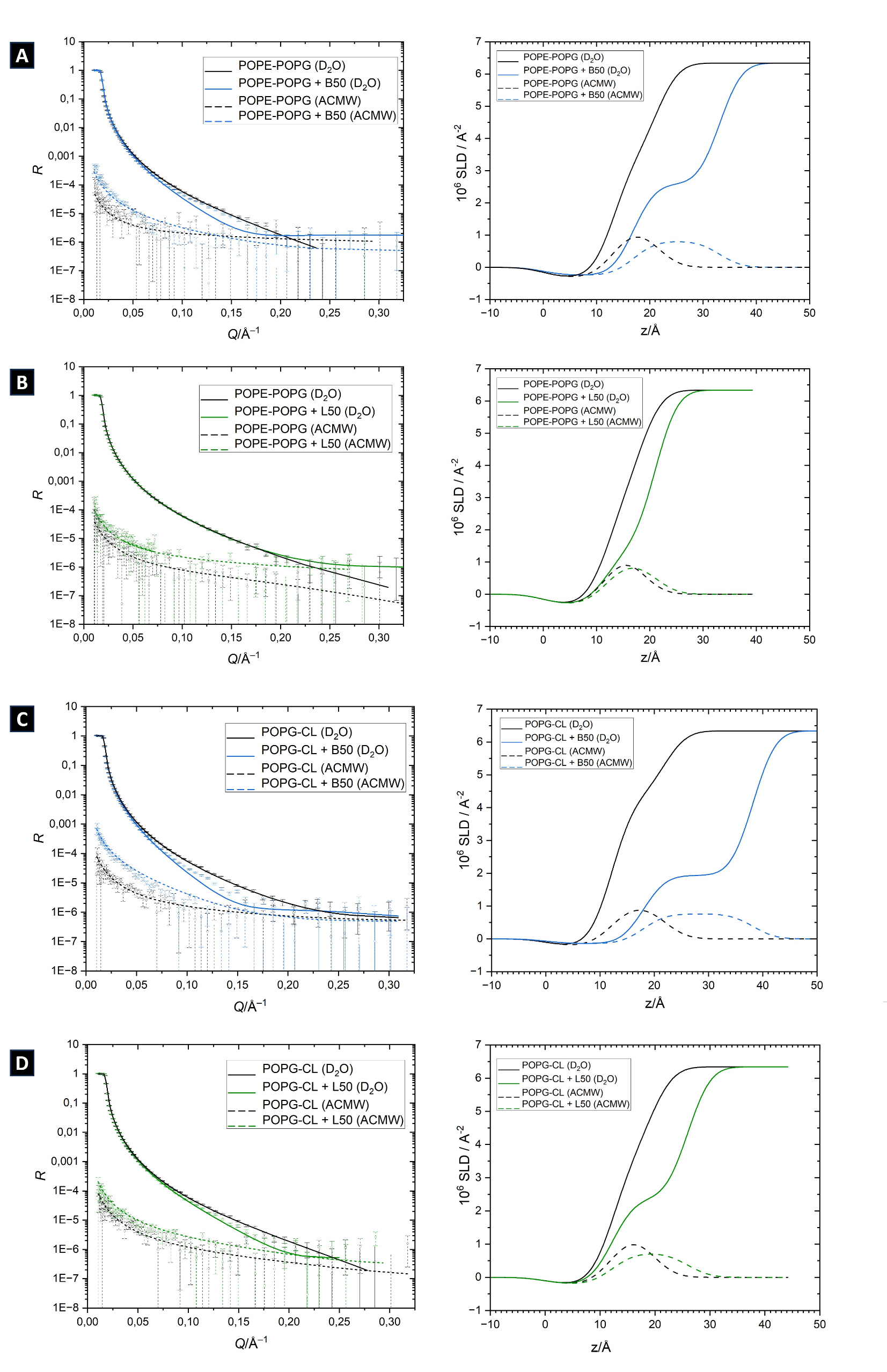


**Figure S7**: NR data (left) obtained for the POPG-CL and POPE-POPG Langmuir monolayers in the absence and presence of the two APs L50 and B50, as shown in the legends: A) POPE-POPG with B50, B) POPE-POPG with L50, C) POPG-CL with BB50, and D) POPG-CL with L50. SLD Profiles (right) obtained though fitting of NR data for the POPG-CL and POPG-POPE Langmuir monolayers in the absence and presence of the two AP polymers L50 and B50. The distance *z* reported on the horizontal axes is measured from the air/hydrophobic layer interface. Solid and dashed lines correspond to fitted data in deuterated water (D_2_O) and air contrast matched water (ACMW) contrasts, respectively.


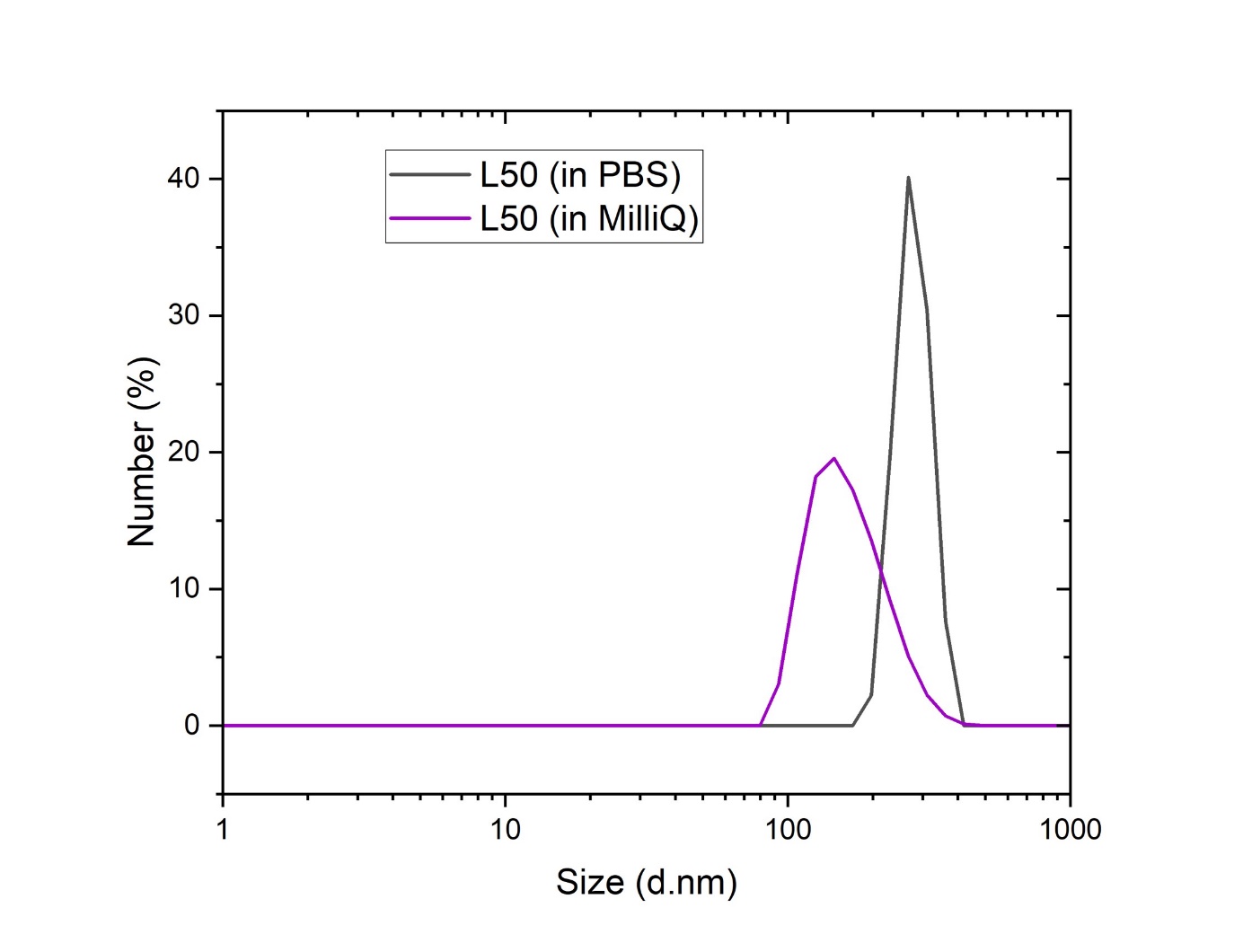


**Figure S8**: DLS distribution by number of L50 in milliQ water and in PBS (1X) buffer indicating formation of self-assembly behavior of linear APs.
